# Development of Multi-Scale X-ray Fluorescence Tomography for Examination of Nanocomposite-Treated Biological Samples

**DOI:** 10.3390/cancers13174497

**Published:** 2021-09-06

**Authors:** Si Chen, Ruben Omar Lastra, Tatjana Paunesku, Olga Antipova, Luxi Li, Junjing Deng, Yanqi Luo, Michael Beau Wanzer, Jelena Popovic, Ya Li, Alexander D. Glasco, Chris Jacobsen, Stefan Vogt, Gayle E. Woloschak

**Affiliations:** 1 X-ray Science Division, Advanced Photon Source, Argonne National Laboratory, Lemont, IL 60439, USA; sichen@anl.gov (S.C.); oantipova@anl.gov (O.A.); luxili@anl.gov (L.L.); junjingdeng@anl.gov (J.D.); yluo89@anl.gov (Y.L.); cjacobsen@anl.gov (C.J.); svogt@anl.gov (S.V.); 2 Department of Radiation Oncology, Feinberg School of Medicine, Northwestern University, Chicago, IL 60611, USA; r-lastra@northwestern.edu (R.O.L.); tpaunesku@northwestern.edu (T.P.); m-wanzer@northwestern.edu (M.B.W.); jelena.popovic@northwestern.edu (J.P.); yali2022@u.northwestern.edu (Y.L.); alexander.glasco@northwestern.edu (A.D.G.); 3 Department of Physics and Astronomy, Weinberg College of Arts and Sciences, Northwestern University, Evanston, IL 60208, USA

**Keywords:** nanoparticles, nanocomposites, X-ray fluorescence microscopy (XFM), X-ray fluorescence (XRF) tomography, cell cycle, BIRC5

## Abstract

**Simple Summary:**

Metal-oxide nanomaterials enter cancer and normal cells even when not specifically targeted, and often interact with specific cellular structures and biological molecules solely due to their innate physical-chemical properties. This raises concerns for the use of nanoparticles, which can be alleviated only with rigorous studies of nanoparticle–cell interactions, studies independent of post-interaction labeling of nanomaterials. X-ray fluorescence microscopy is an imaging technique that quantifies and maps all chemical elements from the periodic table solely based on their native fluorescence excited by the incoming X-ray. We used two different instruments to interrogate the same sample in 3D at two different resolutions and determine heterogeneity of cell-to-cell interactions with nanomaterials, as well as subcellular nanoparticle distribution. This is the first example of multi-scale 3D X-ray fluorescence imaging. This work begins a new era of study on how nanoparticle-based therapies can be developed to be more predictable and safer for use.

**Abstract:**

Research in cancer nanotechnology is entering its third decade, and the need to study interactions between nanomaterials and cells remains urgent. Heterogeneity of nanoparticle uptake by different cells and subcellular compartments represent the greatest obstacles to a full understanding of the entire spectrum of nanomaterials’ effects. In this work, we used flow cytometry to evaluate changes in cell cycle associated with non-targeted nanocomposite uptake by individual cells and cell populations. Analogous single cell and cell population changes in nanocomposite uptake were explored by X-ray fluorescence microscopy (XFM). Very few nanoparticles are visible by optical imaging without labeling, but labeling increases nanoparticle complexity and the risk of modified cellular uptake. XFM can be used to evaluate heterogeneity of nanocomposite uptake by directly imaging the metal atoms present in the metal-oxide nanocomposites under investigation. While XFM mapping has been performed iteratively in 2D with the same sample at different resolutions, this study is the first example of serial tomographic imaging at two different resolutions. A cluster of cells exposed to non-targeted nanocomposites was imaged with a micron-sized beam in 3D. Next, the sample was sectioned for immunohistochemistry as well as a high resolution “zoomed in” X-ray fluorescence (XRF) tomography with 80 nm beam spot size. Multiscale XRF tomography will revolutionize our ability to explore cell-to-cell differences in nanomaterial uptake.

## 1. Introduction

Cellular uptake and subcellular distribution of targeted nanoparticles was shown to correlate with the uptake and distribution of molecules mimicked by the nanoparticles, binding to cell surface proteins such as epidermal growth factor receptor or cell adhesion molecules [1,2]. However, non-targeted nanoparticles can also often have a specific pattern of cellular distribution [3,4]. In our previous work with TiO_2_ nanoparticles or nanocomposites with a TiO_2_ shell, we found that the non-targeted particles enter cells by all active cellular endocytic mechanisms [5,6]. As the metal oxide nanoparticle surface accumulates a complex protein corona [6,7], the behavior of nanocomposites in cells is difficult to predict. Due to the possible benefits of the targeted TiO_2_ shell nanocomposites [8,9], it is desirable to try to uncover the effects of non-targeted nanocomposites in detail, so that they can be monitored or remedied as necessary. In this work, we used the same type of dopamine coated Fe_3_O_4_@TiO_2_ nanocomposites that we have previously evaluated for their capacity to form a protein corona [6]. In this study, we focused on subtle changes in the cell cycle characteristics of HeLa and HCT116 human cancer cells exposed to nanocomposites, and explored the uneven accumulation of nanocomposites in different cells using elemental imaging.

Synchrotron-based X-ray fluorescence microscopy (XFM) is one of the best suited tools to quantitatively study trace element distributions in biological samples and other complex systems. Biologically relevant trace metals, such as iron (Fe), copper (Cu), and zinc (Zn), are measured simultaneously based on their inherent, characteristic X-ray fluorescence (XRF) without the need to add fluorophores for optical microscopy. The elemental sensitivity of the X-ray induced XRF technique is several orders of magnitude better than standard electron beam based XRF due to the absence of bremsstrahlung background. The high penetration power of hard X-ray photons enables studies of biological samples up to a few millimeters thick. To reveal the actual 3D elemental distribution of such a thick volume, XRF tomography has been developed and advanced in various perspectives. Recent studies have presented biological applications of XRF tomography with spatial resolution ranging from a few microns [10,11,12] down to a few hundred nanometers [13,14]. XRF tomography with sub-100 nm spatial resolution has been demonstrated at a few synchrotron-based nanoprobes (e.g., [2,15,16,17]), however with very limited field of view. Combining high spatial resolution and large field of view is conceptually straightforward, however rather challenging in practice with respect to data acquisition and analysis. High spatial resolution X-ray optics is achieved at a cost of efficiency. Substantially more data acquisition time is required, which is not practical at this moment of synchrotron research. Reconstructing a nm-voxel in a volume of hundreds of microns is not trivial due to the so-called self-absorption effect, in which the emitted fluorescence signal is re-absorbed before being detected. Several groups are working to tackle this issue, but no fully developed approach for solving it exists to our knowledge.

To address the need of multi-scale studies, we have demonstrated here a method of multiscale XRF tomography using multiple XFM probes, where a mm-sized sample was scanned, first using a microprobe, followed by secondary sample preparation and subsequent higher-resolution scan of a single cell using a nanoprobe. The ability to perform secondary sample preparation between the measurements using multiple beamlines opens many new opportunities for biological studies. As we demonstrate in this manuscript, a sample that was scanned while paraffin-embedded can be thin sectioned, rehydrated, and used for immunohistochemistry (IHC) and optical microscopy without significant radiation damage and loss of sample quality.

## 2. Materials and Methods

### 2.1. Nanocomposite Preparation

The same batch of nanocomposites used for these experiments was already used for our prior work, and additional details about particle characterization can be found in that publication [6]. Briefly, all chemicals were purchased from Sigma (Sigma-Aldrich Corp., St. Louis, MO, USA) and the nanocomposites were synthesized as described before [2,6,9]. Synthesis of the TiO_2_ shell was conducted in an ice cooled bath by adding TiCl_4_ chilled to −20 °C drop-wise to a diluted colloidal suspension of 2 nm Fe_3_O_4_ nanoparticles; hydrolysis of TiCl_4_ under these conditions favors production of ultrasmall anatase TiO_2_ nanocomposites [18,19] when core particles for shell accumulation are absent. Synthesis of iron oxide nanoparticles was also described previously [2,9]. A combination of FeCl_2_ and FeCl_3_ in 24 mM citric acid was stirred for 3 h at room temperature and aged in static air at 70 °C for 24 h, forming the Fe_3_O_4_ core nanoparticles 1.5 to 3 nm in size. After accumulation of the TiO_2_ shell layer, final nanocomposite sizes varied between 10–20 nm (Figure S1). In the course of synthesis, a color change of nanocomposite suspension from pale rust (pure iron oxide nanoparticle solution) to pale yellow (core-shell nanocomposite solution) was noted; nanocomposite color changed again into brown after application of dopamine surface coating. Changes in UV-visible light spectra and infrared spectra of these nanocomposites were documented in our previous work with this batch of nanocomposites [6]. The final TiO_2_ concentration in colloidal nanocomposite suspension was 200 μg/mL as measured using an X Series II Inductively Coupled Plasma-Mass Spectrometer (Thermo Fisher Scientific, West Palm Beach, FL, USA) at the Northwestern University Quantitative Bio-element Imaging Center (QBIC). A series of standards ranging from 0 ppb to 50 ppb titanium (Ti) was used. All standards and samples were spiked with 3 ppb of indium as an internal control. Using a previously explained approach [9], we calculated that 200 μg/mL of TiO_2_ corresponds to an approximate 180 nM nanocomposite concentration (for a nanocomposite size of 10 nm) with an approximate 300 μM concentration of surface binding sites. The complete TiO_2_ shell surface was covered with dopamine by dissolving 21 mg of powdered dopamine in 55 mL of nanocomposite colloid. The final concentration of dopamine was 2 mM, several fold greater than the molarity of available nanocomposite surface sites. The newly coated nanocomposites were dialyzed in 10 mM sodium phosphate and 40 mM sodium chloride buffer pH ~ 4.5 in order to remove excess dopamine. Use of dialysis tubing (Slide-A-Lyzer™ Dialysis Cassettes, Thermo Fisher Scientific, West Palm Beach, FL, USA) with 2KDal pores allowed for any unbound dopamine be removed from the nanocomposite mixture. In the course of dialysis, the nanocomposite colloidal mixture changed appearance from a transparent, pale-yellow liquid to a partially opaque, light brown solution. As expected, UV-vis absorption showed a red shift at the conclusion of dialysis [20,21]. Nanocomposites stably remained in solution for more than 6 months. Nanocomposites were further diluted in 10 mM sodium chloride, and their zeta potential was found to be −27 mV, as described previously [6].

### 2.2. Cell Culture and Nanocomposite Treatments

Cervical cancer cell line HeLa (CCL-2 ATCC, Manassas, VA, Virginia) was grown in Dulbecco’s Modified Eagle Medium (DMEM), supplemented with 10% fetal bovine serum and 1% penicillin/streptomycin (all obtained from Corning Cellgro, Thermo Fisher Scientific) at 37 °C and 5% CO_2_. Colorectal carcinoma cell line HCT116 (CCL-247 ATCC) was grown in McCoy’s media with 10% fetal bovine serum and 1% penicillin/streptomycin at 37 °C and 5% CO_2_. Mycoplasma testing consisted of optical fluorescence imaging of cells grown on microscope slides and stained with 4′,6-Diamidino-2-Phenylindole, Dihydrochloride (DAPI) diluted in phosphate buffered saline (PBS) (Sigma-Aldrich Corp., St. Louis, MO, USA), similar to the work of others [22].

For flow cytometry, either cell line was seeded in T25 flasks to be 80% confluent at the time of nanocomposite treatment. At the conclusion of the 24 h nanocomposite treatment and a 24 h post-treatment incubation, cells were harvested. A small portion of cells were stained with trypan blue, and the numbers of viable cells counted using a BioRad TC20 (BioRad, Hercules, CA, USA) automated cell counter. The number of HeLa cells per flask averaged at 2 × 10^6^ for nanocomposite treated cells and 4 × 10^6^ for control cells. The numbers of HCT116 cells at 24 h post incubation period were the same as for HeLa cells, while HCT116 cells post-incubated for 48 h numbered 2 × 10^6^ on average in nanocomposite treated samples, and 5 × 10^6^ in control samples. Growth of viable cells into colonies (clonogenic assay) showed that the long-term viability of cells from nanocomposite-treated or control plates was similar for HCT116 cells. Plating efficiency of untreated HCT116 cells was 45 to 53% with the greatest standard deviation (SD) of ± 8.7%, and 34 to 51% with the greatest SD of ± 8.7% for nanoparticle treated cells. In HeLa cells, a minor loss of viability was associated with nanoparticle treatment; plating efficiency of untreated cells was 24 to 28% with the greatest SD of ± 6.4%, while plating of nanoparticle treated cells resulted in growth of 13 to 20% with the greatest SD of ± 8.2% of cells seeded; this difference was significant at *p* = 0.05.

For protein isolation from cells at different density, HeLa cells were plated at 5 × 10^5^ or at 10^6^ cells per T25 flask 16–18 h prior to treatment; these cell densities roughly corresponded to 40% and 80% confluent cells monolayers respectively, at the time of treatment.

For nanocomposite treatments, dialyzed dopamine coated nanocomposites were added as 1/10th of the volume to complete media per T25 flask. The final concentration of TiO_2_ in the media was 20 μg/mL (in standard T25 flasks with 5 mL of media: 4 μg/cm^2^). Each nanocomposite-exposed cell flask was paired with an untreated control flask for all protein and mRNA isolations and flow cytometry assays.

### 2.3. Flow Cytometry

Click-iT^®^ Plus EdU Flow Cytometry Assay Kit (C10635 Life Technologies, Thermo Fisher Scientific, West Palm Beach, FL, USA) was used according to the manufacturer’s instructions to label newly synthesized DNA in cycling cells with EdU (5-ethynyl-2′-deoxyuridine). Cells prepared and treated with nanoparticles as described above were given EdU at a 10 µM concentration during the final hour of post-treatment incubation. Cells were harvested by trypsinization, trypsin was neutralized by addition of complete media and the cells were centrifuged and resuspended in fresh complete media. After removal of a small number of cells for a clonogenic assay, the remaining cells were washed with PBS. Cells were fixed with 4% neutral buffered formalin in PBS for 15 min at room temperature, washed in 1% bovine serum albumin (BSA) in PBS and permeabilized in 1x saponin buffer prepared from the 10x stock provided as a part of the Thermo-Scientific EdU-CLICK kit and 1% BSA in PBS buffer. CLICK labeling with the Alexa-Fluor 647 picolyl azide was performed as recommended and the cells were washed in 1x saponin buffer. Subsequently, cells were incubated with the primary antibody for BIRC5 (MA5-15077, Invitrogen, Waltham, MA, USA), histone H3 (PA5-17869, Invitrogen) or no antibody in 1× saponin buffer for 1 h at room temperature or for 24 h at 4 °C. After a wash in 1% BSA in PBS, test and control cells were incubated with a secondary Alexa-Fluor 488 labeled goat anti-rabbit antibody (A-11034 Thermo Fisher Scientific, West Palm Beach, FL, USA) for 1 h in 1× saponin buffer. Finally, cells were washed in 1% BSA in PBS, and resuspended in PBS with 0.01 mg/mL of 4′,6-Diamidino-2-Phenylindole, Dihydrochloride (DAPI) over night. Samples were processed at the Northwestern University Robert H. Lurie Comprehensive Cancer Center Flow Cytometry Core Facility. A BD LSRFortessa Analyzer (Becton, Dickinson and Company, San Jose, CA, USA) was used, operated at excitation lines at wavelengths of 403 nm, 488 nm, and 640 nm. A typical experimental setup with a labeled control cell sample is shown in Figure S2. Control and nanocomposite treated samples stained only with the secondary antibody showed no false positive events.

### 2.4. Protein Isolation and Western Blots

After nanocomposite treatments of 0, 1, 2, 4, 6, or 24 h, nanocomposite treated and control cells were washed three times with PBS and collected in 100 µL of Radio-Immunoprecipitation-Assay (RIPA) buffer (Thermo Fisher Scientific, West Palm Beach, FL, USA) with Protease inhibitors (Calbiochem, San Diego, CA, USA). Cells were collected by scraping and rocked at 4 °C for 15 min. Cell lysates were centrifuged at 13,000× *g* for 15 min at 4 °C in a tabletop centrifuge (Beckman-Coulter, Indianapolis, IN, USA) to separate proteins from cell debris and the supernatant was transferred to a new microfuge tube. The protein concentration was calculated using Bradford assay with BSA as a standard (NanoDrop 2000, Thermo Fisher Scientific, Waltham, MA, USA). All Bradford assay chemicals came from BioRad (Hercules, CA, USA).

For the isolation of proteins adhering to nanocomposites inside cells, HeLa cells were treated with the nanocomposites in the same way as for “standard” protein isolation above. However, after cell lysis, the supernatant was removed and the pellet was resuspended in 200 µL 2× Laemmli sample buffer (BioRad, Hercules, CA, USA). The resuspended pellet was then centrifuged for 10 min at 10,000× *g*, the supernatant was removed and saved (“the first wash”) and this process was repeated twice more. Finally, the pellet was resuspended in 80 µL of Laemmli sample buffer, boiled at 95 °C for 10 min, spun to remove the nanocomposites, and loaded on a gel the same way as above.

In preparation for Western blots, proteins were diluted with PBS as necessary and mixed with 4× Laemmli Sample Buffer (Bio-Rad). Proteins were then separated on a gradient Sodium Dodecyl Sulfate (SDS) Polyacrylamide Gel (4–20%) (BioRad, Hercules, CA, USA) and transferred to a nitrocellulose membrane (GE Healthcare, Piscataway, NJ, USA). The membrane was blocked by 5% skim milk (Bio-Rad) in 1× TBS-T (Tris-NaCl-Tween 20) for 2 h and then incubated overnight with primary antibodies. This included antibodies against BIRC5 (Ab76424, 1:10,000) and actin (Ab136812, 1:250). The membranes were washed three times with 1× TBS-T, and probed with secondary antibodies (1:10,000) (7074S and 7076S; Cell Signaling, Danvers, MA, USA), tagged with horseradish peroxidase (HRP), and incubated on the blot for 1 h at room temperature. Each membrane was overlaid with Clarity Western Enhanced Chemiluminescence ECL Substrate (BioRad, Hercules, CA, USA) according to the manufacturer’s instructions, and the blots were developed.

### 2.5. HeLa Cell Treatment and Sample Preparation for X-ray Fluoresence Microscopy

Some of the HeLa cells used for flow cytometry were drop-cast onto microporous (2 micron pores, 20 micron pitch) hydrophilic silicon nitride windows (Norcada, Edmonton, AB, Canada). These HeLa cell samples were used to assess distribution of nanocomposites taken up and retained by the cells during a 24 h nanocomposite incubation period followed by a 24 h post-treatment incubation.

To evaluate an early timepoint nanocomposite distribution in our tomography experiment, HeLa cells were grown on Ultralene membrane (Spex, Metuchen, NJ, USA) and treated with nanocomposites for only one hour. The cells were then chemically fixed with 4% formalin and dehydrated. The Ultralene thin film supporting the cells was then rolled into a cylinder-like volume, paraffin embedded, and mounted onto a pin suitable for the tomography rotation stage at the microprobe located at Beamline 2-ID-E at the Advanced Photon Source.

After XRF tomography at 2-ID-E microprobe, secondary sample preparation was performed. The sample was removed from the pin and re-embedded in a larger volume of paraffin. Thin sections of 5 µm thick were produced using a Leica microtome and placed on glass slides in succession, as well as onto silicon nitride (Si_3_N_4_) windows (Silson, Southampton, Warwick, UK). Cell sections on glass slides were processed regularly, using a water bath to assist with attachment of paraffin to the glass; sections lifted onto windows were reduced in size in order to fit the size of the window. The entire volume of the paraffin with the embedded HeLa cells was sectioned. Immunohistochemistry (IHC) was conducted by the Northwestern University Pathology core; BIRC5 primary antibody (71G4B7 Cell Signaling, Danvers, MA, USA) was titrated using control lung tissue sample (Figure S2). Serial IHC images were assembled in a manner that allowed us to identify the position of the section placed on the silicon nitride window. This information was used in combination with the coarse 2D scanning in order to identify a single cell with a high nanocomposite content for high resolution 2D and tomographic XFM imaging.

### 2.6. X-ray Fluorescence Microscopy and Tomography

XFM measurements were performed at the Advanced Photon Source at Argonne National Laboratory, Lemont, IL, USA. The XRF microprobe at Beamline 2-ID-E was used to perform large-field-of-view XRF tomography, and coarse, overview scans of thin sections. The Bionanoprobe (BNP) originally located at Beamline 21-ID-D, now at 9-ID-B, was used for high-resolution tomography of an individual HeLa cell. The two undulator beamlines share very similar design concept. X-ray photons of 10 keV were selected using a double-crystal Si<111> monochromator, then focused using Fresnel zone plates onto a sample. The sample on a pin was raster scanned through the focused X-ray beam, while fluorescence signals were acquired pixel by pixel using an energy-dispersive silicon-drifted detector (Vortex ME-4 Hitachi) located at 90 deg to the incident X-ray beam. The focal spot at the 2-ID-E microprobe was ~600 nm. XRF tomography was performed on the tip of the cylindrical sample. A total of 60 projections were acquired covering a 180 deg angular range with 3 deg rotation step. For each projection, a region of 1.5–1.8 mm in width and 0.4 mm in height was scanned using fly-scan motion (continuous motion in the horizontal direction) with 3 µm step size and 30 msec dwell time per pixel to optimize signal-to-noise ratio and data collection time for the whole set.

High resolution XRF tomography of a sectioned paraffin embedded sample was carried out at the BNP on an individual cell in a thin section attached to a Si_3_N_4_ window. The focal spot was ~80 nm. The tomographic dataset includes a total of 48 projection covering 141 angular range with 3 deg rotation step. With Si_3_N_4_ window, 180 deg angular coverage cannot be achieved as the signal was blocked by the window frame at high rotation angles. Each projection was acquired using fly-scan motion with 80 nm pixel size and 200 ms dwell time per pixel. The scan parameters for tomography data collection are listed in Table 1.

Imaging of the cells from the batch that was used for flow cytometry was conducted at the 2-ID-E beamline with 0.3 micron beam step size; the 2D scan parameters are listed in Table 1.

### 2.7. X-ray Fluorescence Data Analysis and Visualization

Per pixel fluorescence spectra were Gaussian fitted using an in-house developed IDL software, named MAPS [23], which yielded an HDF file for each projection scan. Elemental contents were quantified and calibrated against an AXO standard thin film (AXO DRESDEN GmbH, Dresden, SN, Germany). A recently developed python-based software named XRFtomo [24,25] was used to perform image registration and alignment, mostly using cross-correlation, and normalization, followed by tomographic reconstruction using iterative maximum likelihood problem (PML or PML_hybrid) algorithm in TomoPy. TomoPy was originally developed for absorption-based X-ray computed tomography. Volumetric renderings were generated in Amira (Thermo Fisher Scientific, Waltham, MA, USA).

## 3. Results

### 3.1. Cell Cycle Changes in Cells Exposed to Non-Targeted Nanocomposites

**Figure 1:**
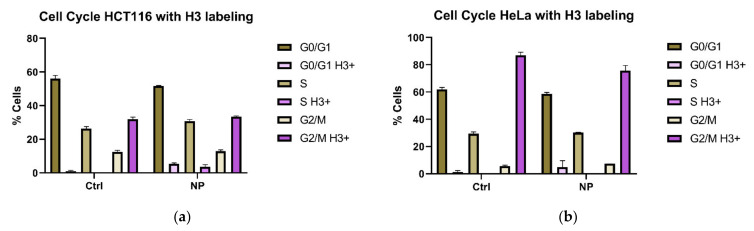
Cell cycle distribution of control and nanocomposite treated cells with H3 labelling. (**a**) Distribution for control and nanocomposite treated HCT116 cells. (**b**) Distribution for control and nanocomposite treated HeLa cells. Analysis for the entire population of cells (≥50,000 per biological replicate) is shown in brown color bars. Pink colored bars, as indicated in the figure legend, apply only to the population of H3 positive cells. Each bar represents the average and standard deviation from triplicate experiments; for gating strategy, see Figure S3; for cell numbers, see Table S1.

Nanocomposites of iron oxide core with titanium dioxide shell and a surface coating of dopamine (Fe_3_O_4_@TiO_2_-DOPA) were prepared as detailed elsewhere [6]; the same batch of nanocomposites was used for this work as well. Nanocomposite sizes varied around 6–10 nanometers (Figure S1), which permits that the outermost TiO_2_ shell layer has a crystal structure that supports stable covalent binding with hydroxyl groups of dopamine [2,6,20,21,26,27]. In our previous work with these non-targeted nanoconstructs at below toxic concentrations, we have found that they accumulate an intracellular protein corona made of a variety of proteins bound to the particle surface with different avidity [6]. In some cases, this led to a temporary protein depletion that lasted as long as 24 h. Considering that these changes in protein availability sufficed to alter gene expression [6], we set out to evaluate whether presence of nanocomposites leads to any differences in cell cycle. Cervical cancer cell line HeLa and colon cancer cell line HCT116 were used for this evaluation. Nanocomposite treatment of either cell line was performed for 24 h, followed by a 24 h incubation period in a nanocomposite free media. During the final hour of the post-nanocomposite treatment, we treated the cells with EdU, and then harvested them. Cell numbers from nanocomposite treated plates were about one half of the numbers harvested from the control-treated plates. However, clonogenic assays of these cells showed no significant differences, regardless of nanocomposite treatment. Only a portion of cells was used for evaluation of viability by clonogenic assay; the remaining cells were fixed and used for cell cycle assessment by flow cytometry. We used a standard strategy to set up flow cytometry gates that allowed us to find the numbers of cells in each phase of the cell cycle (Figure S3). Moreover, in the interest of identifying mitotic cells [28], we have also labeled the cells with the antibodies against phosphorylated histone H3 (Figure 1 and Figure S3). In the remainder of the text, these cells are referred to as H3 positive. Interestingly, we discovered that the distribution of H3 positive cells into different flow cytometry gate regions corresponding to different stages of the cell cycle does not follow the same pattern in the two cell lines used. While in HeLa cells over 80% of all H3 positive cells fell into G2/M gate, as expected, less than 40% of the H3 positive HCT116 cells belonged into G2/M gate (Figure 1). In addition, cell cycle changes after extended, 48 h post-treatment incubation were different from those observed at the 24 h post-incubation timepoint (Figure S4). In nanocomposite treated samples of both HCT116 and Hela cell lines, some of the H3 positive cells could also be found within the S gate. Overall, the changes in the cell numbers within the flow cytometry cell cycle gates in response to nanocomposite treatments in either cell line were subtle (Figure 1, Table S1).

Considering that the total numbers of H3 positive cells in any cell population are few, we expanded our flow cytometry investigation to evaluate a more abundantly expressed cell cycle associated protein–baculoviral inhibitor of apoptosis (IAP) repeat containing protein 5 (BIRC5). BIRC5 is an abundantly expressed protein in both healthy and cancer cells [29]. In mice, knocking out BIRC5 is embryonic lethal. The cells that form in these knockouts are multinucleated, without mitotic spindle and have a catastrophic defect of microtubule assembly [30]. BIRC5 is particularly concentrated in cells actively undergoing mitosis in the G2/M phase of the cell cycle [31,32], although it is present in some quantity in cell nuclei of cancer cells at all times. BIRC5 has an immunohistochemistry staining pattern similar to cancer markers such as Ki67 [33,34]. BIRC5, also known under names apoptosis inhibitor 4 and survivin, is only 142 amino acids in length, and has a single Baculovirus IAP Repeat (BIR) domain and an extended COOH-terminal alpha helix coiled-coil domain [35]. Despite its small size, BIRC5 is involved in numerous cellular processes critical for cell survival and proliferation [31,32,34], acting either as a monomer or as a dimer [36] and cooperating with a large number of other cellular proteins. As BIRC5 engages in numerous protein interactions, and our previous work established that nanocomposite presence perturbs availability of numerous proteins, we repeated the flow cytometry analyses of HeLa and HCT116 cells using antibodies against BIRC5 in the same way as we did for H3.

**Figure 2:**
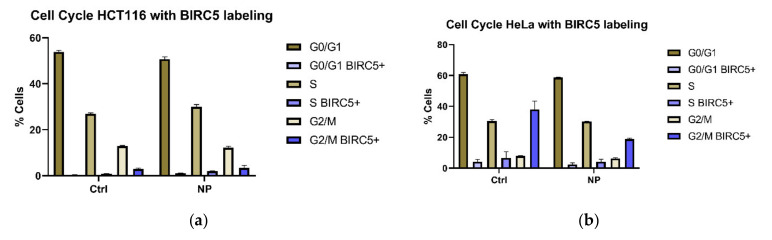
Cell cycle distribution of control and nanocomposite treated cells with BIRC5 labelling. (**a**) Distribution for control and nanocomposite treated HCT116 cells. (**b**) Distribution for control and nanocomposite treated HeLa cells. Analysis for the entire population of cells (≥50,000 per biological replicate) is shown in the brown bars. Purple colored bars, as indicated in the figure legend, apply to BIRC5 positive cells: the most intensely stained ~1000 cells in each case. Each bar represents the average and standard deviation from triplicate experiments; for gating strategy, see Figure S3; for cell numbers, BIRC5 staining intensity, and other details, see Table S1.

In this case, however, cell staining was intense, and we selected the most intensely stained thousand cells from each experiment to evaluate their distribution into different cell cycle gates (Figure S5). The two cell lines used for this analysis showed minor differences in the overall cell cycle cell distribution pattern, but a more marked difference in distribution of BIRC5 positive cells (Figure 2) across the flow cytometry cell cycle gates. In HCT116 cells, numbers of BIRC5 positive cells were the highest in the G2/M phase of the cell cycle, with no significant differences between control and nanoparticle treated cells. In HeLa cells, on the other hand, control cells showed much higher numbers of BIRC5 cells in the G2/M phase of the cell cycle. Median BIRC5 staining intensities in control and nanocomposite treated cells were not different (Table S1). When an extended incubation period with the primary antibody was conducted, the results were almost identical (Figure S6).

### 3.2. BIRC5 Participates in Nanocomposite Protein Corona

**Figure 3:**
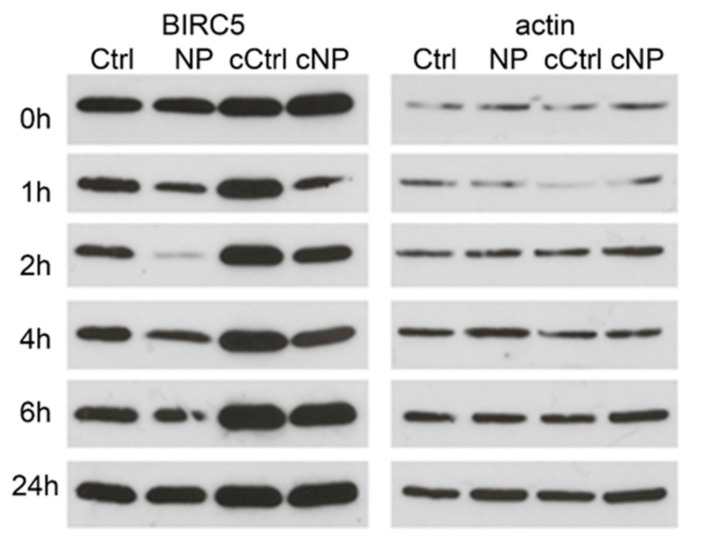
BIRC5 participation in the protein corona on the nanocomposite surface. BIRC5 in whole cell lysates from nanocomposite treated and control cells at different timepoints was evaluated by Western blotting at timepoints indicated. Protein isolates from low cell confluency control (Ctrl) and low cell confluency nanocomposite treated cells (NP) were compared with high confluency control cells (cCtrl) and high confluency nanocomposite treated cells (cNP). Actin protein for the same Western blots is shown on the right-hand side; actin quantity in cells in not affected by cell confluency.

In our prior research, Fe_3_O_4_@TiO_2_-DOPA nanocomposites were found to generate a multi-protein corona after entry into cells [6], including cell surface as well as intracellular proteins. Heat shock protein Hsp90 was found to participate in the outer corona of the nanocomposites; while it was found in proteins eluted from the corona-covered nanocomposites, this elution was complete in the very first wash [6]. Considering that Hsp90 generates protein–protein complexes with BIRC5 and that BIRC5 distribution in HeLa cells was decreased in nanoparticle treated samples, we decided to evaluate whether BIRC5 may be found in the nanocomposite corona. As it is known that BIRC5 expression differs in cells grown at different growth density [37,38,39], we used HeLa cell populations grown to less than 50% or more than 80% confluency. As expected, there was a difference in BIRC5 expression between confluency levels, and the more confluent cells had higher BIRC5 content (Figure 3). HeLa cells grown to both confluency levels were incubated with nanocomposites for different periods of time, from 0 h (nanocomposites were added and the sample processed immediately) to 24 h (nanocomposites were incubated with cells for 24 h and the sample processed without any additional incubation steps). A representative Western blot is shown in Figure 3. In all cases, in sub-confluent and confluent cells alike, addition of nanocomposites leads to an apparent decrease in quantity of BIRC5 by Western blot, especially at 1 and 2 h of incubation (Figure 3). Moreover, evaluation of proteins eluted from nanocomposite pellets (Figure S7) suggests that BIRC5 participates in what is sometimes referred to as “hard” corona-proteins bound to the nanocomposite surface with the greatest permanency. The fact that BIRC5 concentration on regular Western blots markedly recovers when proteins are isolated from samples treated with nanocomposites for longer than 4 h of incubation is more likely due to compensatory protein synthesis than the release of BIRC5 from the nanocomposite bound protein corona.

### 3.3. XFM Permits Imaging of Nanocomposites Inside Cells

We wanted to evaluate the distribution pattern of nanocomposites accumulated in these HeLa cells, so we reserved a small fraction of the cells treated with nanocomposites for 24 h and used them for flow cytometry. While, in most of our elemental cell imaging experiments using XFM, we prepared the cells by growing them directly on silicon nitride windows [2,9,20,26], in this experiment we cast fixed cells onto support. We found that the hydrophilic porous windows permitted the most reliable deposition of fixed cells (Figure S8).

**Figure 4:**
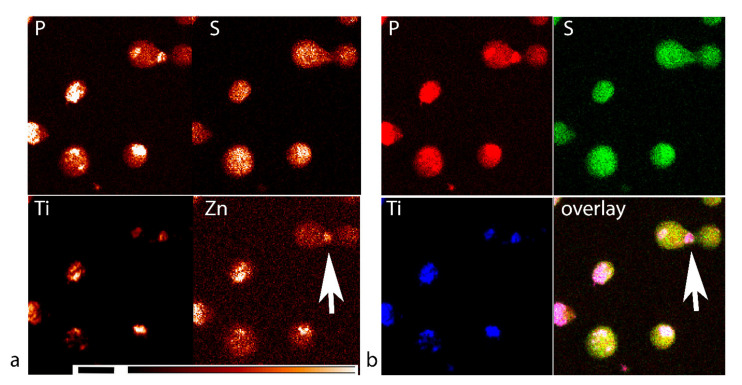
XFM image of HeLa cells treated with nanocomposites for 24 h and incubated post-treatment for 24 h. These cells come from the same batch that was used for flow cytometry shown in Figure 2. (**a**) Red color temperature elemental signal schema with size bar of 20 microns and color bar indicating signal intensity changes from absent–black, to high–white. (**b**) Overlay image for P (red), S (green), and Ti (blue) represent the following: nucleic acids–highest P content, cellular proteins–highest S content, and nanoconjugates–Ti content. Due to the absence of Ti in biological samples, XFM signal-to-background ratio for this element is the strongest. The overlay image shows that distribution patterns of nanoconjugates in the cells vary significantly. The white arrow points to what looks to be a cytokinetic bridge during the telophase, with an increased Zn concentration (red temperature image (**a**)) and overlay of Ti, P, and S signals (three colors overlay image (**b**)).

Patterns of titanium distribution in cells were varied and appeared similar to distribution pattern of BIRC5 [40] in cells at the different stages of the cell cycle. For example, a strong Ti signal indicating distribution of nanocomposites could be seen within what appears to be a cytokinetic bridge (white arrows in Figure 4, showing stronger Zn signal in the bridge between two cells and colocalization of Ti signal with P and S signals). Other cellular distribution patterns that resemble BIRC5 distribution in other stages of the cell cycle were noticeable as well (Figure 4). Nevertheless, without a dual evaluation of nanocomposite distribution and staining for BIRC5, it is impossible to be certain that the nanocomposites in the cells pointed by the white arrow co-localize with a cytokinetic bridge due to interaction with BIRC5.

### 3.4. Use of X-ray Fluorescence Tomography to Evaluate Heterogeneity of Nanocomposite Distribution in Cell Clusters and Cells by “Low-Resolution Tomography” and “High-Resolution Tomography”

**Figure 5:**
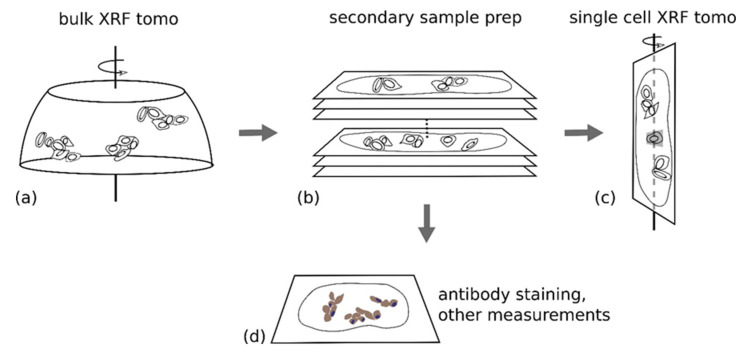
Schematic showing the workflow of multiscale tomography. (**a**) Paraffin embedded cell clusters supported on Ultralene thin film were examined by performing XRF tomography with coarse resolution; (**b**) The sample was sectioned into a series of 5 µm thick slices; (**c**) A single cell (highlighted in gray) on one of the thin sections was again examined by performing XRF tomography, but with fine resolution (80 nm pixel size for 2D projections); (**d**) Antibody staining was performed on other thin slices, which were then imaged using visible light. This served a dual purpose of confirming that IHC after XFM of the bulk imaged sample is possible and to generate a 3D model of the cell cluster so that we can establish the position of the single cell imaged in (**c**) inside the entire cluster imaged in (**a**).

One of the questions that is implicitly raised by this work is: is there a threshold for nanocomposite uptake by individual cells that will result in the changed cell cycle? As mentioned before, fluorescent labeling of nanocomposite prior to treatment could change their behavior or be potentially unstable (e.g., [9,41]). Due to this, an evaluation of nanocomposites, based on their elemental content, is a better approach to gain an answer to this question. While bulk techniques for evaluation of elemental concentration, such as inductively coupled plasma mass spectrometry, can be calculated on a per cell basis, such evaluation would only provide the average nanocomposite content of the cells. The very best way to evaluate nanocomposite uptake by individual cells is to do a high throughput evaluation of elemental content XFM of single cells. Nevertheless, XFM has rarely been used to evaluate elemental content of a large numbers of cells, either in cell culture experiments or when working with animal tissues (Figure S9). This has interfered with our ability to establish the range of nanocomposite uptake on a per cell basis. An ideal solution for this would be to image cell clusters tomographically in 3D, and then further select representative single cells from the imaged volume to conduct high resolution single cell tomography. A schematic presentation of work is shown in Figure 5. It should be noted that our approach is unique both due to the secondary sample preparation and the independently completed tomography on two different instruments as detailed in Methods Section 2.5 and Section 2.6. By comparison, work by others who conducted serial tomography [42] involved imaging of the same area of the same sample, without additional processing; while tomograms differed only by numbers of imaging angles used.

**Figure 6:**
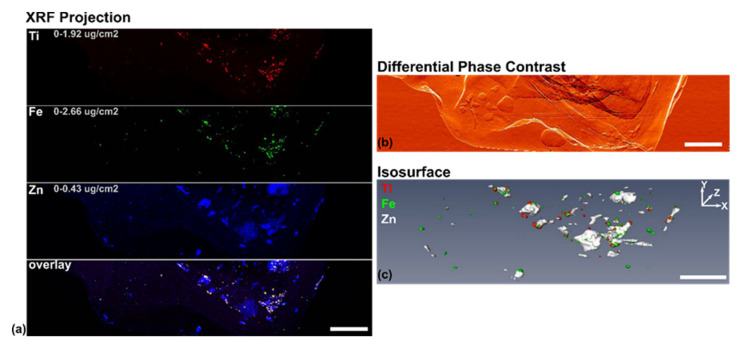
XRF projection and reconstructed volume of the sample. (**a**) 2D XRF projection of the sample, showing the distributions of Ti, Fe, Zn, and their overlay from the cells; (**b**) 2D differential phase contrast image of the sample, with the structures mainly formed by paraffin and Ultralene thin film; (**c**) Isosurface of reconstructed 3D volume (see rotation movie Video S1: outerTomo) showing the distributions of both the cell, represented by Zn (white) and the nanocomposites, represented by Ti (red) and Fe (green). The scale bar in each case is 200 µm.

XFM imaging at a series of rotation angles was conducted and the 3D reconstruction of the sample generated. A series of 2D projections, such the one as presented in Figure 6, was used to generate a movie of sample rotation (Video S1: outerTomo). Isosurface of this 3D reconstruction is shown in Figure 6c.

Overall elemental content of the cell cluster and the variance of elemental per pixel concentrations are provided in Table S2. Even at this level of overview information, we can easily realize the significant differences between cells with regard to the nanocomposite content. For example, while the total quantity of phosphorus in the entire sample (representing predominantly DNA content of the cells) is 5965 femtograms, quantities of Ti and Fe are an order of magnitude lower at 867 and 663 fg, respectively. Considering that the sample self-absorption for a sample thickness used is significant and greatest for P > Ti > Fe; this cumulative fg measurement should be considered provisional at this time. However, regardless of the total amounts of the three elements, their standard deviation for pixel-to-pixel differences was approximately the same at: 1.5, 1.5, and 1 fg for P, Ti, and Fe, respectively. This shows that the heterogeneity of nanocomposite content between cells is an order of magnitude greater than variation which can be expected for native biological elements.

**Figure 7:**
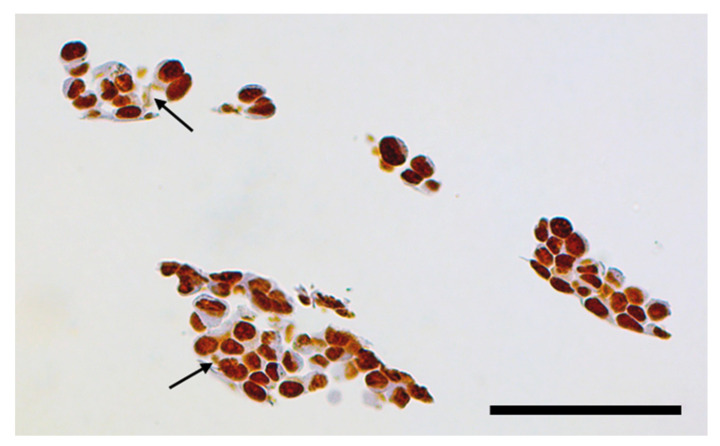
Immunohistochemistry for BIRC5 shows typically stained cell nuclei (for a control tissue sample see Figure S2). However, in addition to cell nuclei, some lightly stained material is also noticeable in the cytosol of many cells (e.g., areas indicated by arrows), possibly containing aggregates of nanocomposites with BIRC5 attached to particle surface in a manner permitting its recognition by the antibodies. Black bar corresponds to 100 microns.

**Figure 8:**
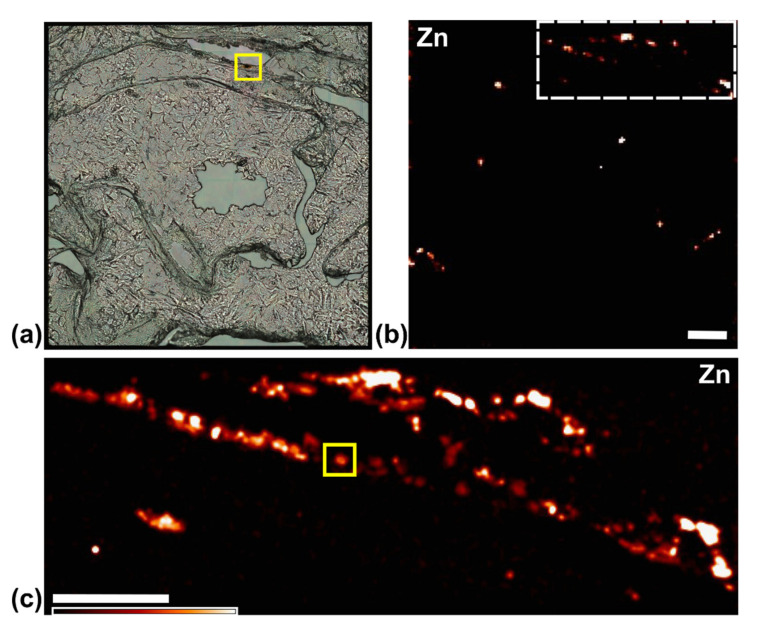
Overview images of a 5 µm-thick slice from the cell cluster sample on an XFM compatible “window” with 1.5 × 1.5 mm^2^ Si_3_N_4_ film. (**a**) Visible light image of the entire window; (**b**) 2D fluorescence mapping of the entire window showing the cell distribution, represented by Zn signal; the rectangle region indicated by white dashed lines was zoomed in by another 2D scan with smaller pixel size, as Zn signal shown in (**c**). The yellow squares in both (**a**,**c**) indicated the same single cell, which was later on imaged tomographically in 3D at the BNP instrument. Color sale bar goes from no signal (black) to highest signal (white). The scale bars in (b) and (c) are 200 µm.

**Figure 9:**
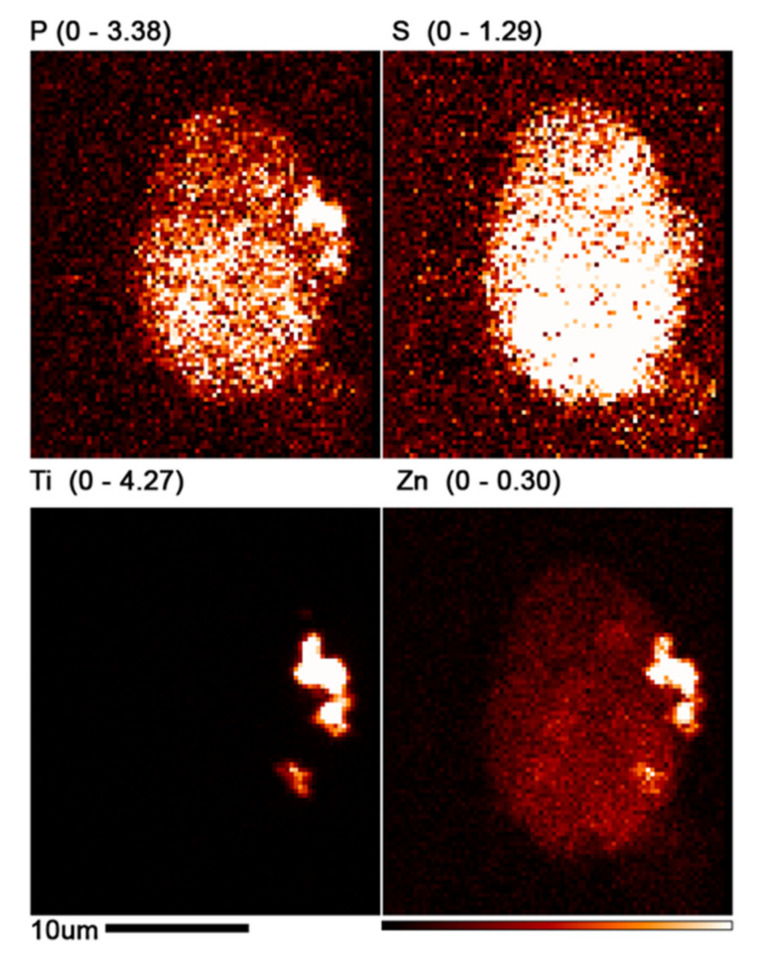
High resolution XFM scan of the cell selected in Figure 8. The map for P suggests that the cell nucleus is most likely in the bottom half of the cell, while the map for Ti indicates that the nanocomposite aggregates remain in cytosol. The scale bar is 10 microns. The minimal and maximal values (in μg/cm^2^) of the color bar for each element are shown next to the elemental symbol, with black pixels corresponding to the minimal values. (For example, for Ti, black pixels correspond to 0 μg/cm^2^; white pixels correspond to concentrations of at least 4.27 μg/cm^2^).

**Figure 10:**
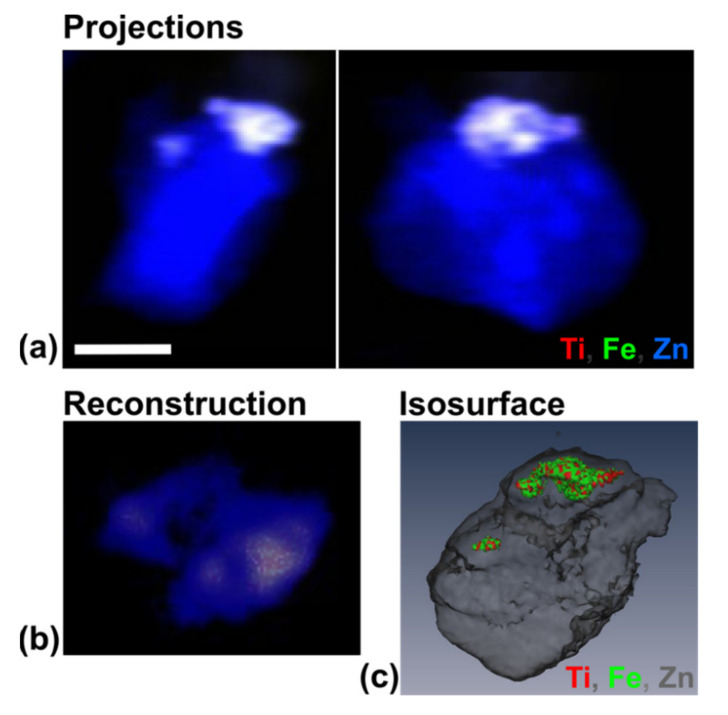
XRF tomography of a single cell performed at the BNP. (**a**) 2D projections of the cell from two different rotation angles, showing the overlay of the cell (represented by Zn in blue) and the nanocomposites (represented by Ti in red and Fe in green; consequently, nanocomposite rich region appears white); (**b**) a single reconstructed cross section showing the overlay of Ti, Fe, and Zn; (**c**) Isosurface of reconstructed 3D volume of the cell (Zn in gray) with the nanocomposites (Ti in red and Zn in green) inside. For the rotation movie of this sample, see Video S1: innerTomo. The scale bar is 5 µm.

Following tomographic imaging at the 2-ID-E microprobe, paraffin embedded sample was encased in a larger volume of paraffin and sectioned. Individual tissue sections were either placed on glass slides for immunohistochemistry (IHC) for BIRC5 (Figure 7) or on silicon nitride windows for 2D XFM imaging (Figure 8 and Figure 9) and high-resolution tomography (Figure 10). For the rotation movie of this sample, see Video S2: innerTomo.

## 4. Discussion

This study demonstrates the versatility of XFM for two-stage tomographic imaging of nanocomposite treated cells at different resolutions. A high-throughput analysis of cells treated with dopamine coated Fe_3_O_4_@TiO_2_ nanocomposites demonstrated that the nanocomposite distribution in cells is highly heterogeneous. One of the nanocomposite-containing cells from the same pre-imaged sample was selected for high-resolution tomographic imaging for determination of subcellular nanocomposite distribution. One hour after beginning treatment, nanocomposites were clustered in two large aggregate clusters within the cell. To generate this image, we conducted low resolution XRF tomography, sectioned the sample after tomography, and prepared sample sections suitable for IHC as well as for a high-resolution XRF tomography of single cells. The ability to perform secondary sample preparation after the initial XRF tomography for visible light microscopy (VLM) and high-resolution XFM evaluation opens many new opportunities for biological studies.

The possibility to conduct evaluation of nanocomposite uptake on the level of tissues using the approach shown here will be critical for translational and biomedical research. For example, one may be able to correlate different tissue cell types (established by IHC conducted after XRF tomography as we have, here) with different nanocomposite uptake metrics. Moreover, the ability to select a subregion of the sample for additional high resolution XFM will allow us to determine whether nanocomposite uptake in different types of cells may be associated with different intracellular structures. This type of “imaging scaling” where the same sample can be evaluated in a high-throughput manner, followed by high resolution imaging that informs about subcellular structures, is impossible with any other technique that can be used for nanocomposite visualization.

Changes in the distribution of BIRC5 in cells in different stages of the cell cycle, coupled with the DNA-maintenance alterations in nanocomposite-treated cells can be expected to lead to genomic instability in cells exposed to nanocomposites in concentrations below toxic levels. This is in keeping with findings of others who noted increased numbers of cells with micronuclei after exposure to TiO_2_ nanoparticles [43]. However, while most of the TiO_2_ work so far has focused exclusively on oxidative stress and its role in genotoxicity and cytotoxicity, this study shows that many additional issues need to be considered in nanocomposite work and that fluctuations of the content of nanoparticle protein corona have to be investigated in detail for any nanocomposite work to be complete.

In our previous work with dopamine coated core-shell nanocomposites, we found heat shock protein Hsp90 as one of the proteins that participates in a transient cytoplasmic protein corona on the nanocomposite surface [6]. Additionally, in other prior research we found that proteins that attach to the nanocomposite surface may maintain their interactions with other proteins [2,7]. In this study, we found that BIRC5 participates in the nanocomposite corona. As one of the well-established pro-cancer interactions of BIRC5 is its binding with the Hsp90 [44], we now propose that it is likely that this protein–protein interaction is preserved while BIRC5 participates in the protein corona of nanocomposites. By extension, we also suggest that it is possible that other interactions of BIRC5 may also occur while it is attached to the nanocomposites. At the same time, it is also possible that some of the BIRC5 interactions are made impossible by its interaction with the nanocomposites. Decreased availability of BIRC5 is known to cause pleiotropic cell-division defects [31,45] and its presence is the most critical during the G2/M phase of the cell cycle [33]. Dynamics of BIRC5 expression in HeLa cells have been studied in great detail [30,31,32,44,46,47,48,49,50,51,52,53,54,55,56,57,58,59,60,61]. Its overexpression stabilizes microtubules against nocodazole-induced depolymerization [52], while its suppression increases numbers of polyploid and micronucleated cells [30,31,55]. BIRC5 is a critical component of the chromosome passage protein complex necessary for chromosome alignment and segregation during mitosis and cytokinesis [30,46,52,53,55,59,61]. Moreover, BIRC5 is also transported to mitochondria [50,51,57] and prevents apoptosis by inhibition of caspases 3 and 7 [36,38]. Finally, BIRC5 is found in cancer-derived exosomes, where it may play a role in disease progression [56,58]. It will be valuable to replicate some of these studies in cells treated with nanocomposites that bind BIRC5, and evaluate the extent of its change in function caused by binding to the nanocomposite surface.

## 5. Conclusions

Nanocomposite treatment in this study was conducted at a concentration, that was not overly toxic as in the past [6]. Nevertheless, the actual concentration of nanocomposites in individual cells may vary significantly compared to an average nanocomposite concentration. Elemental imaging of cells and cell clusters (Figure 4 and Figure 6) shows a dramatic cell-to-cell variation with respect to nanocomposite uptake, as well as subcellular nanocomposite distribution pattern. If retention of nanoconjugates in cells is driven by their protein corona, it is highly likely that the subcellular distribution patterns of non-targeted nanoconjugates taken up by the cells will coincide with the “regular” subcellular distribution of proteins that they have accumulated within the corona. This is a very important consideration for work with the non-targeted nanoconstructs, as well as targeted nanoconstructs that may perhaps lose their targeting moieties. This study is too limited in scope to establish answers to the questions we pose. However, it is doubtless that XRF tomography-within-tomography will play a significant role in generating accurate and meaningful answers to questions pertaining to nanoparticle retention inside cells.

In addition, we have shown that samples imaged by XFM can be post-processed for IHC with BIRC5 being our example. While we still must do IHC on exactly the same silicone nitride widows scanned by XFM in order to provide the final confirmation that nanocomposites colocalize with BIRC5, imaging of single cells analogous to work shown in Figure 4 may provide us with additional proofs for this possibility. BIRC5 plays numerous roles in carcinogenesis and metastasis, and has therefore been targeted by different small molecule, antibody, and nanocomposite therapies, both on its own or through its binding with Hsp90 [54,56,62,63,64,65,66,67,68,69]. In this work, we found that non-targeted dopamine-coated Fe_3_O_4_@TiO_2_ nanocomposites have a high affinity for BIRC5, making it one of the proteins of the “hard” protein corona. Future studies should evaluate whether this interaction can be used to interfere with the cell cycle in a controlled manner as a part of novel cancer therapies.

## Figures and Tables

**Table 1:** Data collection parameters.

Tomography Data Collection Parameters						
Beamline	Incident X-ray Energy (Kev)	Flux (Counts/s)	Dwell/Pixel (ms)	Step Size (mm)	# Projections	Angular Coverage (°)
2-ID-E	10	~10^9^	30	3	60	180
Bionanoprobe	10	3 × 10^9^	200	0.08	48	141
**2D Scan Parameters**						
**Beamline**	**Incident X-ray Energy (Kev)**	**Flux** **(Counts/s)**	**Dwell/Pixel** **(ms)**	**Step Size** **(mm)**		
2-ID-E	10	~10^9^	50	0.3		

The top panel contains scan parameters at Beamline 2-ID-E and the BNP for coarse and fine tomography data collection, respectively and the bottom panel contains scan parameters used at Beamline 2-ID-E for 2D scans of the cells that were used for flow cytometry. #-corresponds to the number of sample projection images collected for tomographic data reconstruction.

## Data Availability

Any data referred to in this work will be available on request.

## Supplementary Materials

The following are available online at https://www.mdpi.com/article/10.3390/cancers13174497/s1, Figure S1: High resolution image of core-shell nanocomposites, Figure S2: Control immunohistochemistry (IHC) staining for BIRC5 (brown) with hematoxylin counterstain for cell nuclei (blue), Figure S3: Cell cycle distribution evaluation by flow cytometry, Figure S4: Cell cycle flow cytometry of nanocomposite treated HCT116 cells with different post-treatment incubation times, Figure S5: Cell cycle distribution of cells with the highest BIRC5 expression, Figure S6: Cell cycle evaluation of nanoparticle treated and control cells and the cells with the highest BIRC5 expression, Figure S7: Elution of BIRC5 from nanoparticles, Figure S8: XFM map of two cell pairs, Figure S9. XFM map of a tissue sample from a rabbit treated with core-shell nanocomposites, Table S1: Flow cytometry data obtained for HCT116 and HeLa cells exposed to nanocomposites for 24 h and post-incubated without nanocomposites for 24 h, Table S2. Elemental content analysis of cells imaged by low-resolution XRF tomography, Video S1: outerTomo, Video S2: innerTomo.
