# Supplementary material for: Development of Multi-Scale X-ray Fluorescence Tomography for Examination of Nanocomposite-Treated Biological Samples
Source: Cancers (Basel). 2021 Sep 6;13(17):4497. doi: 10.3390/cancers13174497 (PMC8430782; doi:10.3390/cancers13174497)
Supplement: Supplementary file 1 [file cancers-13-04497-s001.zip › Supplementary materials.pdf]

# Supplementary Material: Development of Multi-Scale X-Ray Fluorescence Tomography for Examination of Nanocomposite-Treated Biological Samples

**Si Chen, R. Omar Lastra, Tatjana Paunesku, Olga Antipova, Luxi Li, Junjing Deng, Yanqi Luo, M. Beau Wanzer, Jelena Popovic, Ya Li, Alexander D Glasco, Chris Jacobsen, Stefan Vogt and Gayle Woloschak**

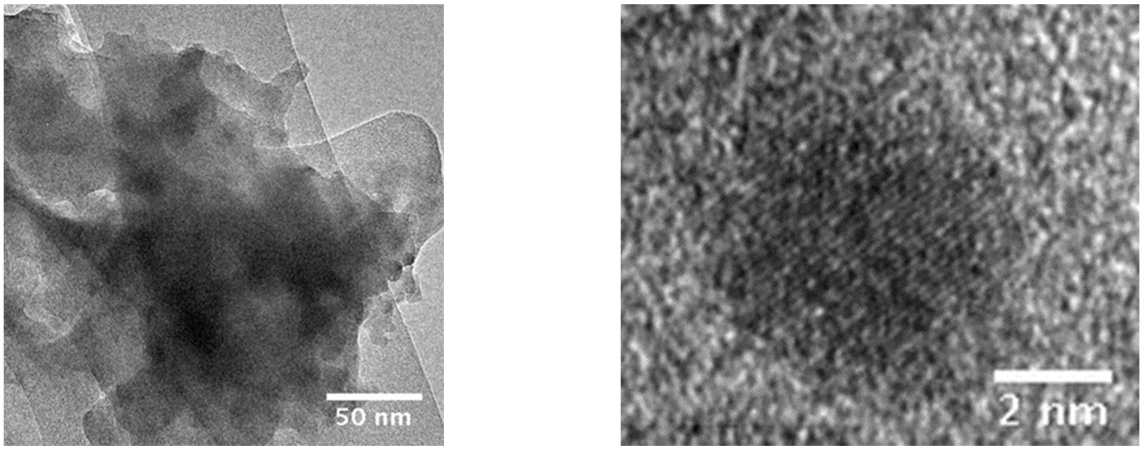


**Figure S1.** High resolution image of core-shell nanocomposites. Transmission Electron Microscopy images of $Fe_3O_4$@$TiO_2$ nanocomposites coated with dopamine ($Fe_3O_4$@$TiO_2$-DOPA) acquired at the NUANCE TEM facility, Northwestern University, courtesy of Dr. Paul Smeets.

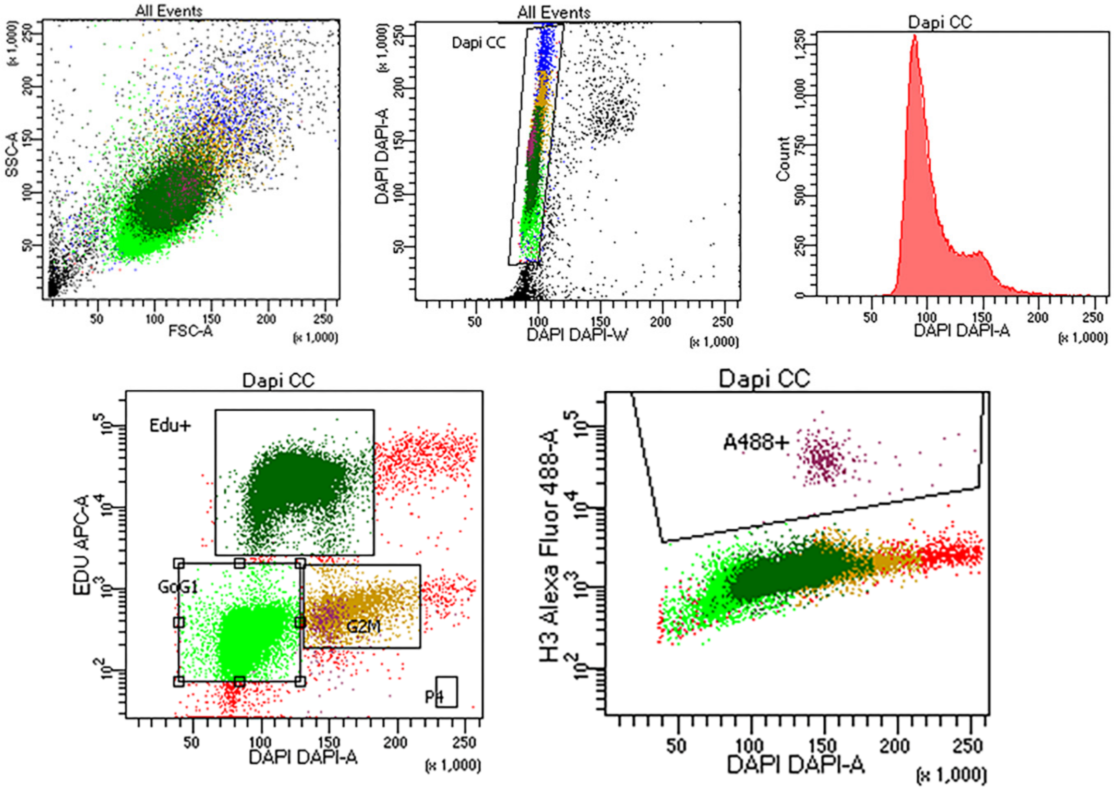


**Figure S2.** Cell cycle distribution evaluation by flow cytometry. Cellular DNA was stained with DAPI after CLICK- labeling of EdU incorporated into DNA over a period of one hour. Please note that plotting cells by their DAPI Area vs. Width

allowed us to separate cell singlets (Gate “Dapi CC”) from cell aggregates. This gating strategy allowed us to use only cell singlets for dot plots shown in the lower panels and develop gating strategy for cells in G0/G1, S (EdU+) or G2/M stage of the cell cycle. Finally – gating (Gate A488+) used for selection of H3 positive cells is also shown.

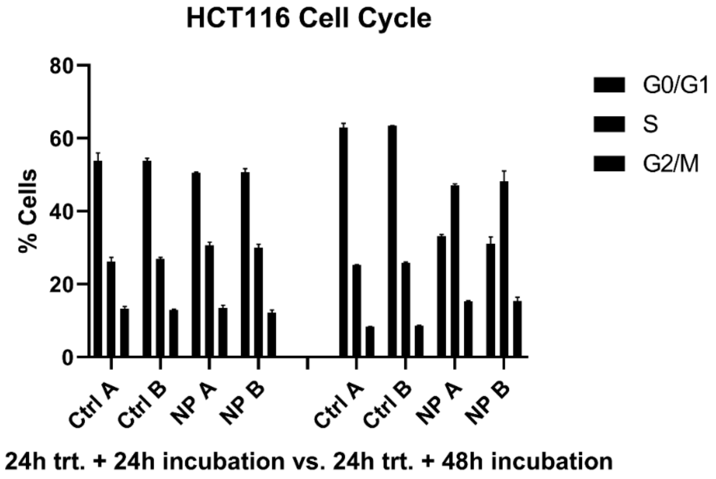


**Figure S3.** Cell cycle flow cytometry of nanocomposite treated HCT116 cells with different post-treatment incubation times. Cells were incubated for 24 or 48 hours in nanocomposite free media after the initial 24h long nanocomposite treatment. Data from three biological replicates, with standard deviation, is presented in each case. A and B labels indicate separate experiments.

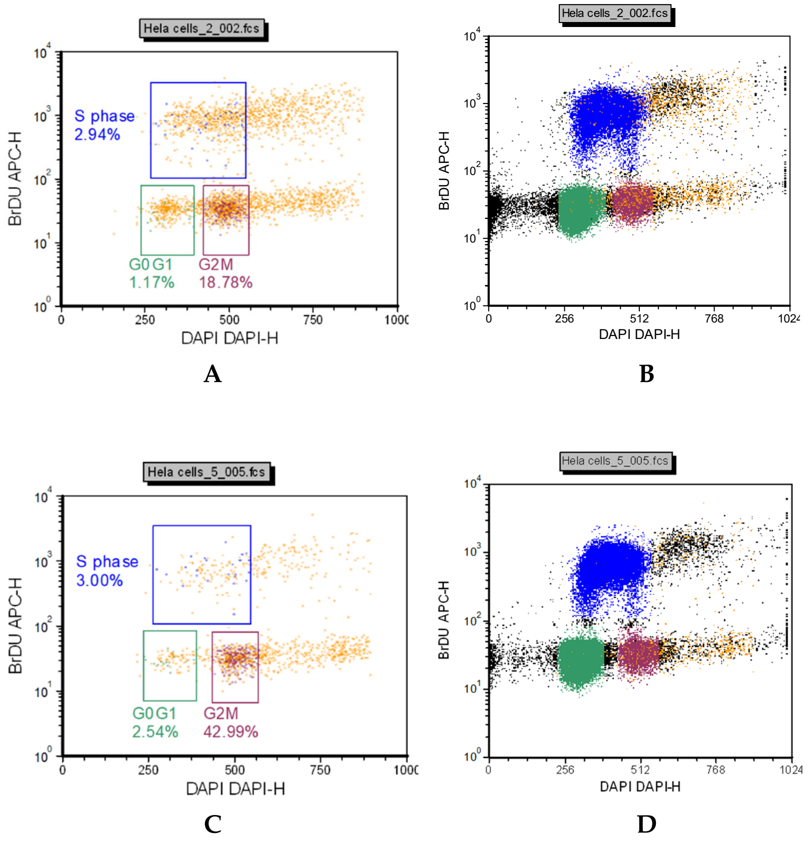


**Figure S4.** Cell cycle distribution of cells with the highest BIRC5 expression. About 1000 brightest BIRC5 positive (panels A and C) and all ~60,000 (panels D and D) HeLa cells examined in separate flow cytometry experiments. In nanocomposite

treated samples (A and B), majority of cells with the highest BIRC5 content are found in cells that do not fit into the cell cycle gates (for exact cell numbers see Table S1). In control cells (C and D) distribution of the most brightly stained BIRC5 positive cells matches cell cycle gates better (for additional discussion see Figure 2.).

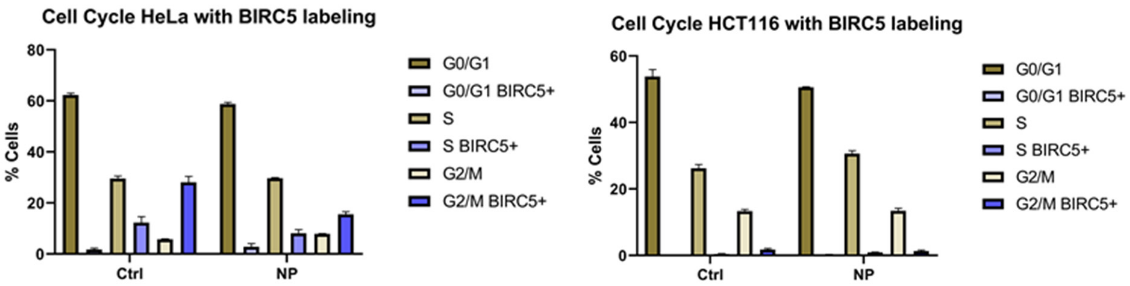


**Figure S5.** Cell cycle evaluation of nanoparticle treated and control cells and the cells with the highest BIRC5 expression. Replicate of experiments from Figure 2 with an extended, 24h at 4°C incubation with the primary BIRC5 antibody. No significant differences in results are noticeable for either cell line. For exact numbers of cells see Supplemental Table 1.

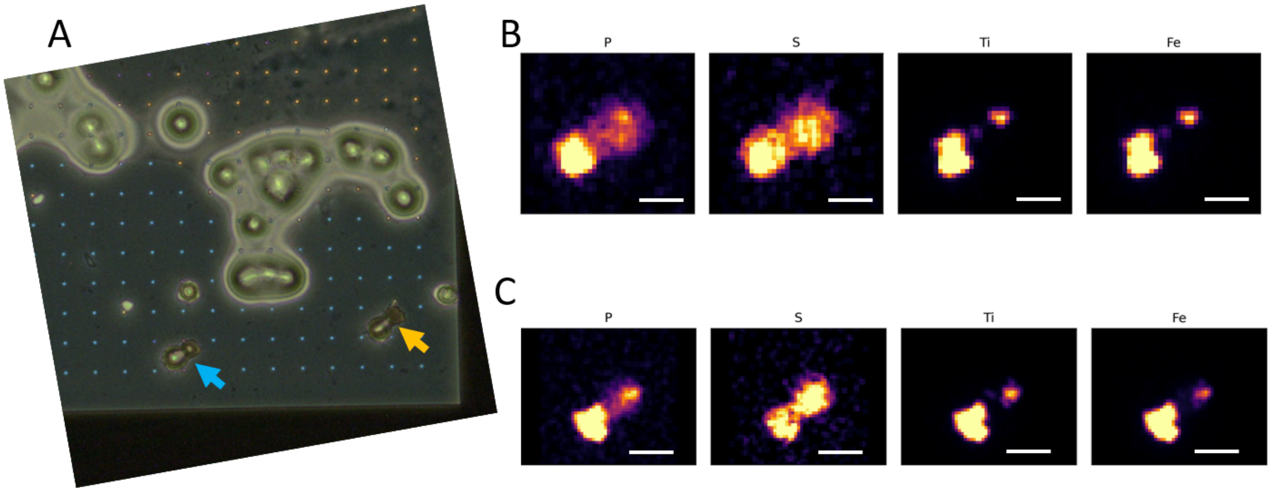


**Figure S6.** XFM of two cell pairs. A. visible light image of a silicon nitride window with HeLa cells; B. cell pair indicated by the blue arrow; C. cell pair indicated by the yellow arrow. Scale bar 10 micron. Elemental imaging by XFM was done with cells used for flow cytometry, treated with nanocomposites for 24h and post-incubated in nanocomposite-free media for 24h. Here two cells apparently in the process of cell division were imaged, in each case showing poorly executed nuclear division: P signal, corresponding to cell nucleus, is disproportionate in two parts of the cell. Sulfur signal, corresponding to cellular proteins is more equally distributed in the two nascent daughter cells. Titanium (Ti) and iron (Fe) signals, indicating nanocomposite presence inside the cell are prominent and equally disproportionate in portions of the cells close to the nucleus. It is probable that in these two examples presence of the nanocomposites hampered normal BIRC5 in the process of mitosis and lead to disproportional cell division.

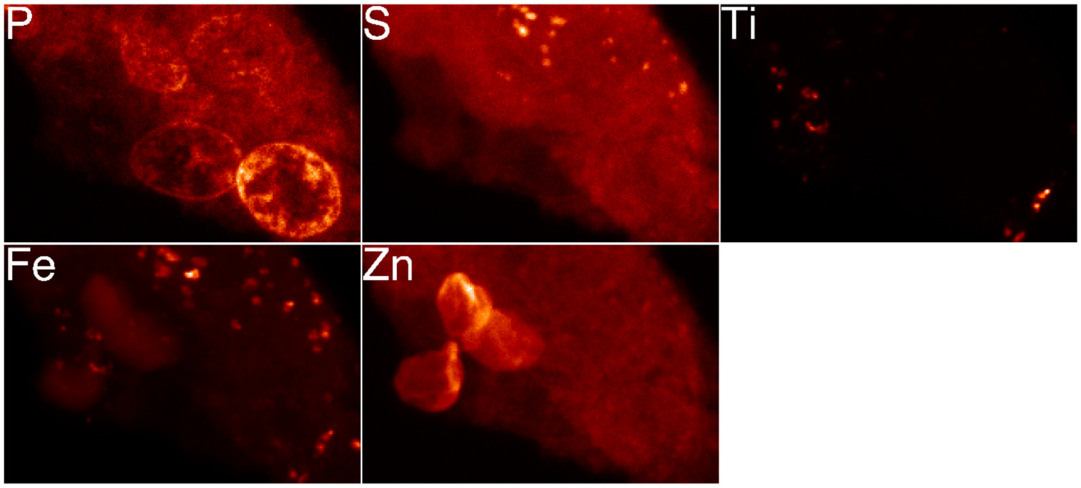


**Figure S7.** XFM maps of a tissue sample from a rabbit treated with core-shell nanocomposites. Tissue sample originates from a previous study [23]. Elemental maps obtained for phosphorus (P), sulfur (S), titanium (Ti), iron (Fe) and zinc (Zn) each show different aspects of cells. P map shows cell nuclei most clearly because of the high P content in genomic DNA, Zn signal outlines red blood cells most clearly, while Ti shows locations of nanocomposites. This image of a 50 x 30 micron area contains only a few cells (only four cell nuclei appear in this image) which is insufficient for any type of high-throughput analysis.

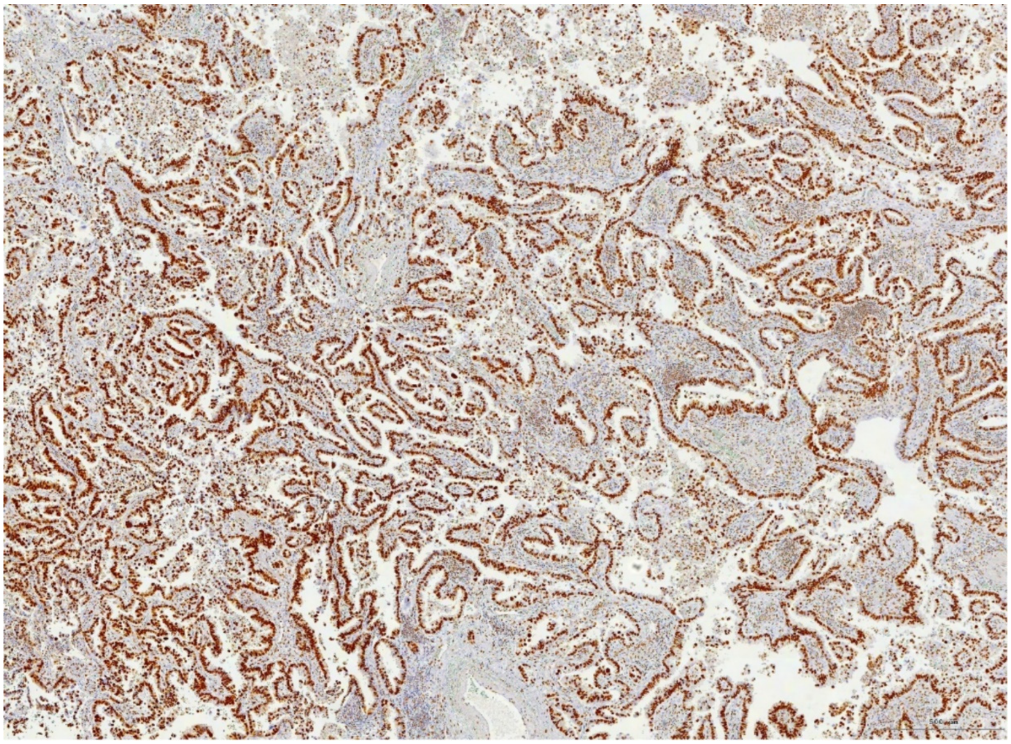

**Figure S8.** Control immunohistochemistry (IHC) staining for BIRC5 (brown) with hematoxylin counterstain for cell nuclei (blue). A control lung tissue sample shows characteristic BIRC5 staining of cell nuclei. The same antibody dilution and IHC conditions were used for staining of paraffin-embedded and IHC-stained HeLa cells.

**Table S1.** Flow cytometry data obtained for HCT116 and HeLa cells exposed to nanocomposites for 24h and post-incubated without nanocomposites for 24h. Cell counts for H3 positive and Birc5 positive cells and their gate distribution are also indicated.

| | | - | | | % | % | % | % | % | % |
|---|---|---|---|---|---|---|---|---|---|---|
| **HeLa** | **NP** | **Singlets** | **H3 Positive** | **Median** | **H3G0G1** | **H3S** | **H3G2M** | **G0G1** | **S** | **G2M** |
| 7 | 24+24 | 59398 | 305 | 1715.44 | 1.08 | 0 | 78.76 | 59.83 | 29.75 | 7.31 |

| | | | | | | | | | | |
|---|---|---|---|---|---|---|---|---|---|---|
| 8 | 24+24 | 58805 | 241 | 1485.51 | 3.1 | 0 | 76.9 | 58.32 | 30.63 | 7.27 |
| 9 | 24+24 | 60465 | 128 | 1213.36 | 10.2 | 0 | 71.43 | 57.64 | 29.91 | 7.23 |
| ave | | 59556 | 224.6667 | 1471.437 | 4.793333 | 0 | 75.69667 | 58.59667 | 30.09667 | 7.27 |
| sd | | 841.2033 | 89.62329 | 251.3357 | 4.790003 | 0 | 3.81028 | 1.120907 | 0.468757 | 0.04 |
| 10 | ctrl | 60093 | 316 | 1498.93 | 0.29 | 0 | 89.18 | 63.52 | 27.91 | 6.36 |
| 11 | ctrl | 59908 | 271 | 1274.88 | 0.67 | 0 | 86.91 | 60.63 | 30.51 | 5.34 |
| 12 | ctrl | 59893 | 208 | 1309.75 | 2.55 | 0 | 84.68 | 61.58 | 29.78 | 5.35 |
| ave | | 59964.67 | 265 | 1361.187 | 1.17 | 0 | 86.92333 | 61.91 | 29.4 | 5.683333 |
| sd | | 111.3927 | 54.24942 | 120.5566 | 1.210124 | 0 | 2.25003 | 1.47299 | 1.341007 | 0.586032 |
| | | - | | | % | % | % | % | % | % |
| **HCT116** | **NP** | **Singlets** | **H3 Positive** | **Median** | **H3G0G1** | **H3S** | **H3G2M** | **G0G1** | **S** | **G2M** |
| 19 | 24+24 | 59257 | 247 | 1000 | 4.63 | 2.49 | 33.1 | 51.47 | 31.68 | 12.3 |
| 20 | 24+24 | 59284 | 288 | 991.05 | 5.14 | 3.05 | 33.92 | 51.9 | 29.41 | 13.79 |
| 21 | 24+24 | 59037 | 381 | 842.91 | 6.04 | 5.06 | 33.42 | 51.88 | 30.78 | 12.61 |
| ave | | 59192.67 | 305.3333 | 944.6533 | 5.27 | 3.533333 | 33.48 | 51.75 | 30.62333 | 12.9 |
| sd | | 135.4855 | 68.661 | 88.22588 | 0.713933 | 1.351456 | 0.41328 | 0.242693 | 1.143081 | 0.786193 |
| 22 | ctrl | 59350 | 217 | 1104 | 0.33 | 0 | 32.51 | 54.3 | 27.42 | 13.32 |
| 23 | ctrl | 59326 | 441 | 930.57 | 0.83 | 0 | 30.38 | 55.41 | 26.51 | 12.81 |
| 24 | ctrl | 59301 | 419 | 1046 | 1.53 | 0.09 | 32.76 | 58.15 | 24.76 | 11.66 |
| ave | | 59325.67 | 359 | 1026.857 | 0.896667 | 0.03 | 31.88333 | 55.95333 | 26.23 | 12.59667 |
| sd | | 24.5017 | 123.4666 | 88.28557 | 0.602771 | 0.051962 | 1.307912 | 1.351925 | 1.351925 | 0.850314 |
| | | - | | | % | % | % | % | % | % |
| **HeLa** | **NP** | **Singlets** | **Birc5 Positive** | **Median** | **BircG0G1** | **BircS** | **BircG2M** | **G0G1** | **S** | **G2M** |
| 13 | 24+24 | 60412 | 1177 | 1186.37 | 1.48 | 6.44 | 16.23 | 59.54 | 29.39 | 7.8 |
| 14 | 24+24 | 60781 | 1133 | 1263.46 | 2.97 | 8.69 | 14.46 | 58.53 | 29.91 | 8 |
| 15 | 24+24 | 61059 | 1183 | 1175.74 | 4.03 | 9.21 | 16.08 | 58.14 | 29.76 | 7.91 |
| ave | | 60750.67 | 1164.333 | 1208.523 | 2.826667 | 8.113333 | 15.59 | 58.73667 | 29.68667 | 7.903333 |
| sd | | 324.5648 | 27.30079 | 47.87251 | 1.281028 | 1.472288 | 0.981478 | 0.722519 | 0.267644 | 0.100167 |
| 16 | ctrl | 59980 | 1157 | 1345.57 | 2.26 | 11.92 | 30.3 | 62.95 | 28.72 | 5.99 |
| 17 | ctrl | 59942 | 1127 | 1382.37 | 1.2 | 10.1 | 28.31 | 61.47 | 30.65 | 5.72 |
| 18 | ctrl | 60010 | 1058 | 1309.75 | 1.88 | 14.81 | 25.63 | 62.41 | 29.38 | 5.6 |
| ave | | 59977.33 | 1114 | 1345.897 | 1.78 | 12.27667 | 28.08 | 62.27667 | 29.58333 | 5.77 |
| sd | | 34.07834 | 50.76416 | 36.3111 | 0.537028 | 2.37517 | 2.34348 | 0.748955 | 0.980935 | 0.19975 |
| | | - | | | % | % | % | % | % | % |
| **HeLa** | **NP** | **Singlets** | **Birc5 Positive** | **Median** | **BircG0G1** | **BircS** | **BircG2M** | **G0G1** | **S** | **G2M** |
| 2 | 24+24 | 60333 | 1154 | 632.09 | 1.41 | 3.18 | 19.52 | 59.09 | 30.07 | 7.92 |
| 3 | 24+24 | 60808 | 1030 | 655.25 | 2.57 | 3 | 18.81 | 58.2 | 30.41 | 8.09 |
| 4 | 24+24 | 60814 | 1099 | 667.14 | 3.4 | 6.08 | 18.18 | 58.51 | 30.01 | 7.55 |
| ave | | 60651.67 | 1094.333 | 651.4933 | 2.46 | 4.086667 | 18.83667 | 58.6 | 30.16333 | 7.853333 |
| sd | | 275.9897 | 62.13158 | 17.82442 | 0.99955 | 1.728622 | 0.670398 | 0.451774 | 0.215716 | 0.276104 |
| 5 | ctrl | 60006 | 1052 | 557.31 | 3.74 | 3.92 | 42.22 | 60.83 | 29.73 | 6.72 |
| 6 | ctrl | 59985 | 1033 | 518.61 | 2.79 | 4.65 | 39.92 | 59.68 | 31.5 | 6.26 |
| 7 | ctrl | 60035 | 1186 | 441.09 | 5.85 | 11.25 | 32.17 | 62 | 30.09 | 5.69 |
| ave | | 60008.67 | 1090.333 | 505.67 | 4.126667 | 6.606667 | 38.10333 | 60.83667 | 30.44 | 6.223333 |
| sd | | 25.10644 | 83.39265 | 59.1807 | 1.566216 | 4.037776 | 5.265533 | 1.160014 | 0.935468 | 0.515978 |
| | | - | | | % | % | % | % | % | % |
| **HCT116** | **NP** | **Singlets** | **Birc5 Positive** | **Median** | **BircG0G1** | **BircS** | **BircG2M** | **G0G1** | **S** | **G2M** |
| 25 | 24+24 | 59958 | 1139 | 973.38 | 0.3 | 1.05 | 1.38 | 50.71 | 31.23 | 12.91 |
| 26 | 24+24 | 59961 | 1091 | 1084.32 | 0.29 | 0.86 | 2.06 | 50.67 | 29.64 | 13.98 |
| 27 | 24+24 | 59886 | 1029 | 1084.32 | 0.22 | 0.85 | 1.85 | 50.33 | 31.07 | 13.08 |
| ave | | 59935 | 1086.333 | 1047.34 | 0.27 | 0.92 | 1.763333 | 50.57 | 30.64667 | 13.32333 |
| sd | | 42.46175 | 55.14828 | 64.05124 | 0.043589 | 0.112694 | 0.348186 | 0.208806 | 0.875462 | 0.575007 |
| 28 | ctrl | 59825 | 1050 | 1018.15 | 0.07 | 0.38 | 1.03 | 52.92 | 26.71 | 13.63 |
| 29 | ctrl | 59726 | 1020 | 1074.61 | 0.06 | 0.32 | 1.51 | 52.26 | 27.01 | 14.1 |
| 30 | ctrl | 59687 | 1017 | 1018.15 | 0.19 | 0.57 | 1.48 | 56.25 | 24.89 | 12.56 |
| ave | | 59746 | 1029 | 1036.97 | 0.106667 | 0.423333 | 1.34 | 53.84333 | 26.20333 | 13.43 |
| sd | | 71.14071 | 18.24829 | 32.5972 | 0.072342 | 0.130512 | 0.268887 | 2.102958 | 1.147229 | 0.78924 |
| | | - | | | % | % | % | % | % | % |
| **HCT116** | **NP** | **Singlets** | **Birc5 Positive** | **Median** | **BircG0G1** | **BircS** | **BircG2M** | **G0G1** | **S** | **G2M** |
| 9 | 24+24 | 59809 | 868 | 495.81 | 0.93 | 1.98 | 2.33 | 50.38 | 30.63 | 11.44 |
| 10 | 24+24 | 59837 | 982 | 491.37 | 1.09 | 2.16 | 3.61 | 51.8 | 28.79 | 12.8 |
| 11 | 24+24 | 59755 | 1045 | 649.38 | 1.05 | 1.69 | 4.4 | 49.97 | 30.44 | 12.37 |

| | | | | | | | | | | |
|---|---|---|---|---|---|---|---|---|---|---|
| ave | | 59800.33 | 965 | 545.52 | 1.023333 | 1.943333 | 3.446667 | 50.71667 | 29.95333 | 12.20333 |
| sd | | 41.68133 | 89.71622 | 89.97279 | 0.083267 | 0.237136 | 1.044621 | 0.96033 | 1.011945 | 0.69515 |
| 12 | ctrl | 60047 | 988 | 461.38 | 0.3 | 0.9 | 2.48 | 54.02 | 27.37 | 12.82 |
| 13 | ctrl | 60002 | 1003 | 385.42 | 0.24 | 0.61 | 3.36 | 53.05 | 26.99 | 13.23 |
| 14 | ctrl | 59908 | 945 | 486.97 | 0.56 | 0.78 | 2.97 | 54.39 | 26.63 | 12.76 |
| ave | | 59985.67 | 978.6667 | 444.59 | 0.366667 | 0.763333 | 2.936667 | 53.82 | 26.99667 | 12.93667 |
| sd | | 70.92484 | 30.10537 | 52.81599 | 0.170098 | 0.145717 | 0.440946 | 0.692026 | 0.370045 | 0.255799 |

**Table S2.** Elemental content analysis of cells imaged by low-resolution XFM tomography. Elemental content data for region of interest (ROI) containing cells and a cell-free, background elemental content area of the XFM map. Yellow color indicates elemental content and standard deviation values for phosphorus (P), titanium (Ti), and iron (Fe) in the cell-rich area of the sample.

| **Element** | **Sample** | | | | | | **Background** | **SAMPLE Final** |
|---|---|---|---|---|---|---|---|---|
| **[ug/cm2]** | **mean** | **max** | **min** | **total content [mean x scan area 179.976 um²] in femtograms** | **stddev** | **stddev_radius2** | **mean** | **total content after background subtraction in femtograms** |
| P | 3.574912 | 9.897832 | 0.718388 | 6433.9935 | 1.459632 | 0.2314843 | 0.26045351 | 5965.230532 |
| S | 1.969058 | 3.543763 | 0.274301 | 3543.836 | 0.648095 | 0.1157958 | 0.10513417 | 3354.614652 |
| Cl | 1.002963 | 1.265109 | 0.656989 | 1805.0944 | 0.068389 | 0.0591285 | 1.106446 | -186.245464 |
| K | 0.035977 | 0.38575 | 0.002873 | 64.750333 | 0.040865 | 0.0082809 | 0.021010102 | 26.93709971 |
| Ca | 0.415252 | 1.6914 | 0.075254 | 747.35568 | 0.204168 | 0.0272032 | 0.019603046 | 712.0738274 |
| Ti | 0.487431 | 10.22307 | 0.002462 | 877.25994 | 1.455468 | 0.0621187 | 0.005778466 | 866.8588206 |
| Mn | 0.003141 | 0.015936 | 6.16E-15 | 5.6532063 | 0.001496 | 0.0011624 | 0.001290773 | 3.330116265 |
| Fe | 0.372218 | 7.526634 | 0.006445 | 669.90414 | 1.045601 | 0.0449004 | 0.004012758 | 662.6811752 |
| Cu | 0.010637 | 0.063827 | 0.003559 | 19.14323 | 0.006703 | 0.0014359 | 0.006169 | 8.040483953 |
| Zn | 0.095398 | 0.464102 | 0.019604 | 171.69401 | 0.058979 | 0.0059512 | 0.003023071 | 166.2529635 |
