# Supplementary material for: Development of Multi-Scale X-ray Fluorescence Tomography for Examination of Nanocomposite-Treated Biological Samples
Source: Cancers (Basel). 2021 Sep 6;13(17):4497. doi: 10.3390/cancers13174497 (PMC8430782; doi:10.3390/cancers13174497)
Supplement: Supplementary file 1 [file cancers-13-04497-s001.zip › Western Blot Information/Nanocomposites and cell lysates 12-9.pdf]

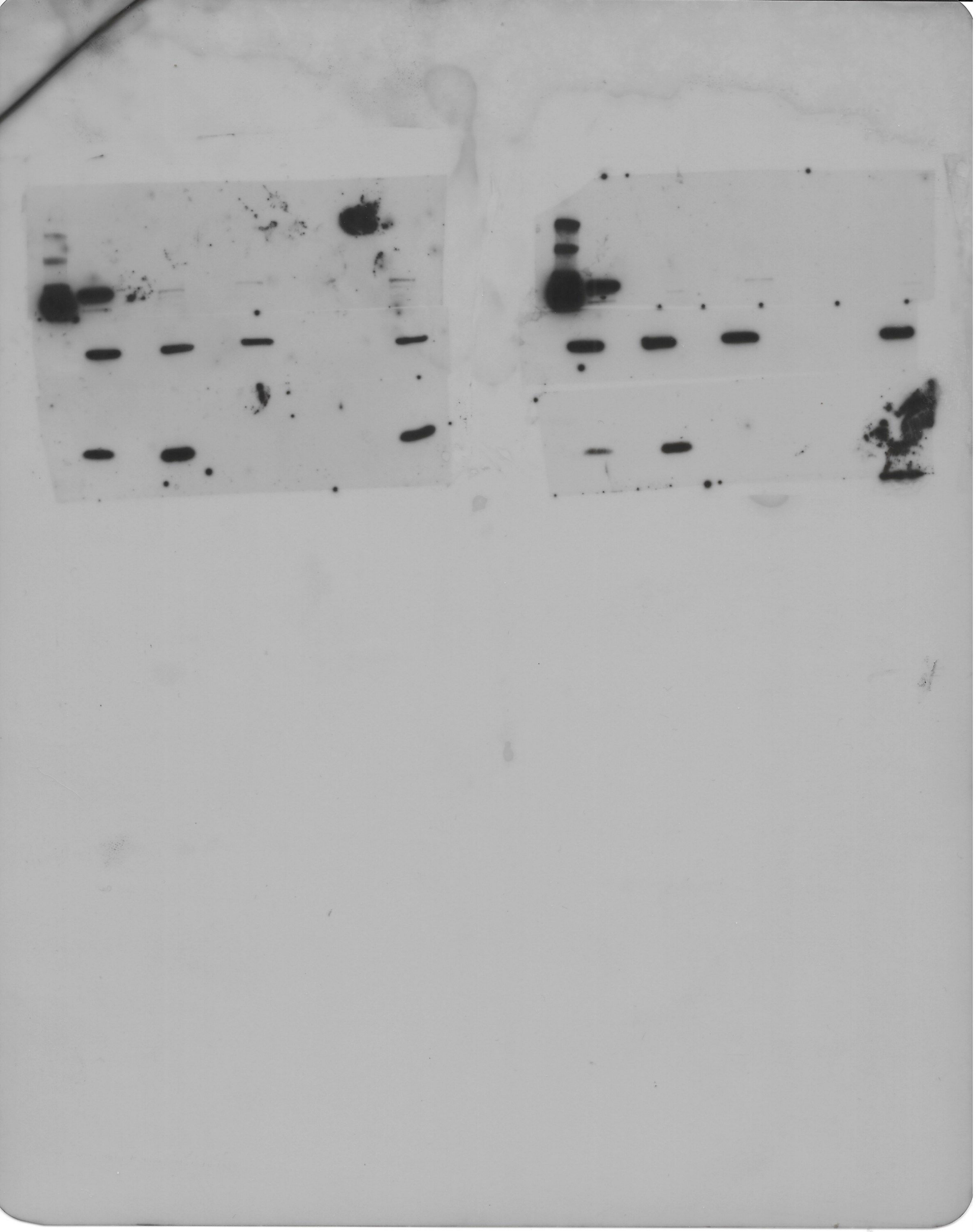

Proteins from cell lysate "as is" and accumulating on nanoconstruct surface

WB strips:
Hsp90 (top)
Actin (middle)
BIRC5 (bottom)

Order of lanes:
marker
input lysate
0
eluate from nanocomposite
0
supernatant after first spin
wash 1
wash 2
wash 3
eluate fron nanocomposite (repeat)
