# Supplementary material for: Development of Multi-Scale X-ray Fluorescence Tomography for Examination of Nanocomposite-Treated Biological Samples
Source: Cancers (Basel). 2021 Sep 6;13(17):4497. doi: 10.3390/cancers13174497 (PMC8430782; doi:10.3390/cancers13174497)
Supplement: Supplementary file 1 [file cancers-13-04497-s001.zip › Western Blot Information/Nanoparticles in cells 4h.pdf]

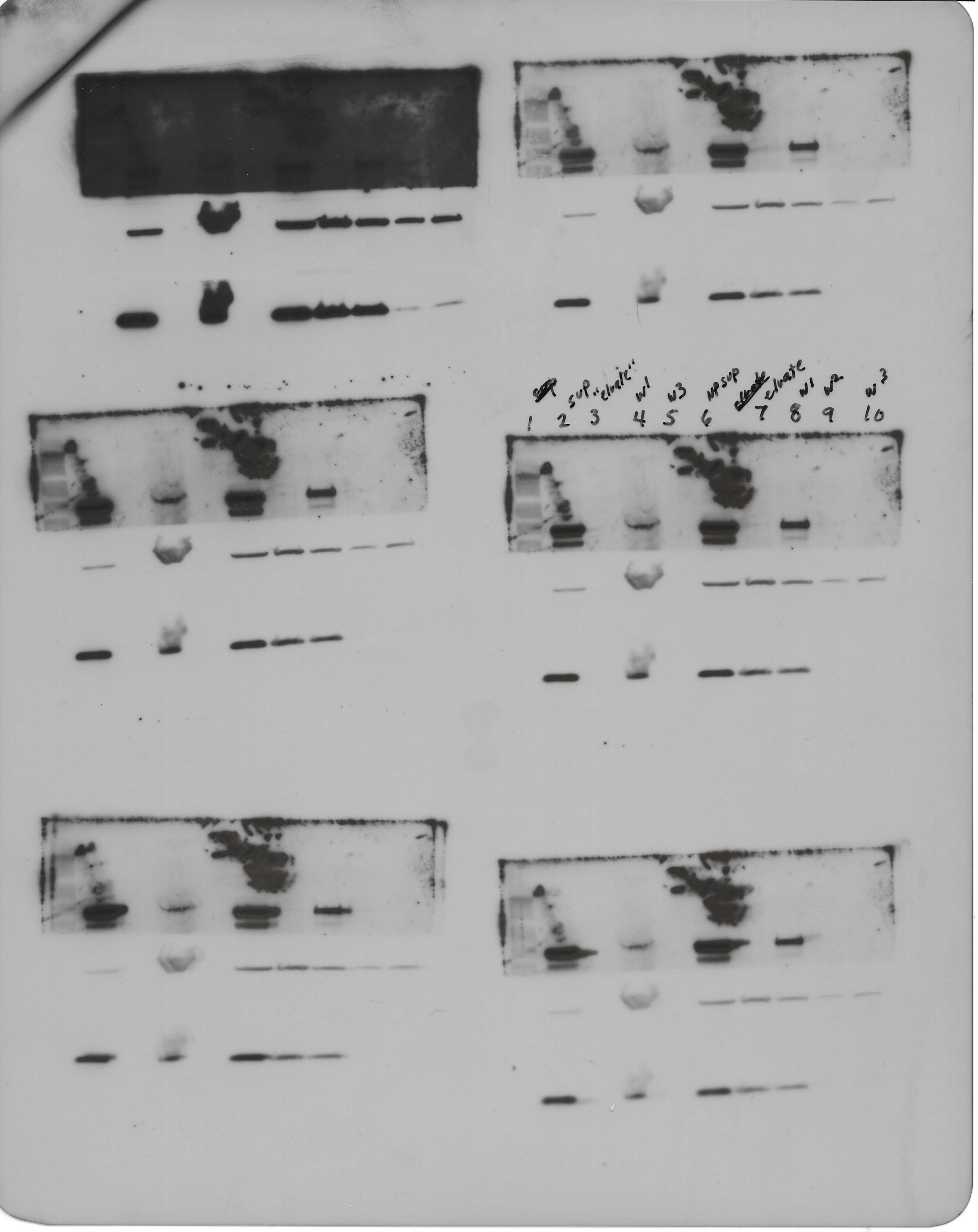

Nanoparticles applied to cells for 4h

Three rows corespond to different segments of the same WB membrane; size difference permits imaging of all three a tthe same time:
Hsp90
Actin
BIRC5

Order of lanes:
Marker
Control SN
Control eluate
Control wash 1
Control wash 3
NP sample SN
NP sample eluate
NP sample wash 1
NP sample wash 2
NP sample wash 3
