# Supplementary material for: Development of Multi-Scale X-ray Fluorescence Tomography for Examination of Nanocomposite-Treated Biological Samples
Source: Cancers (Basel). 2021 Sep 6;13(17):4497. doi: 10.3390/cancers13174497 (PMC8430782; doi:10.3390/cancers13174497)

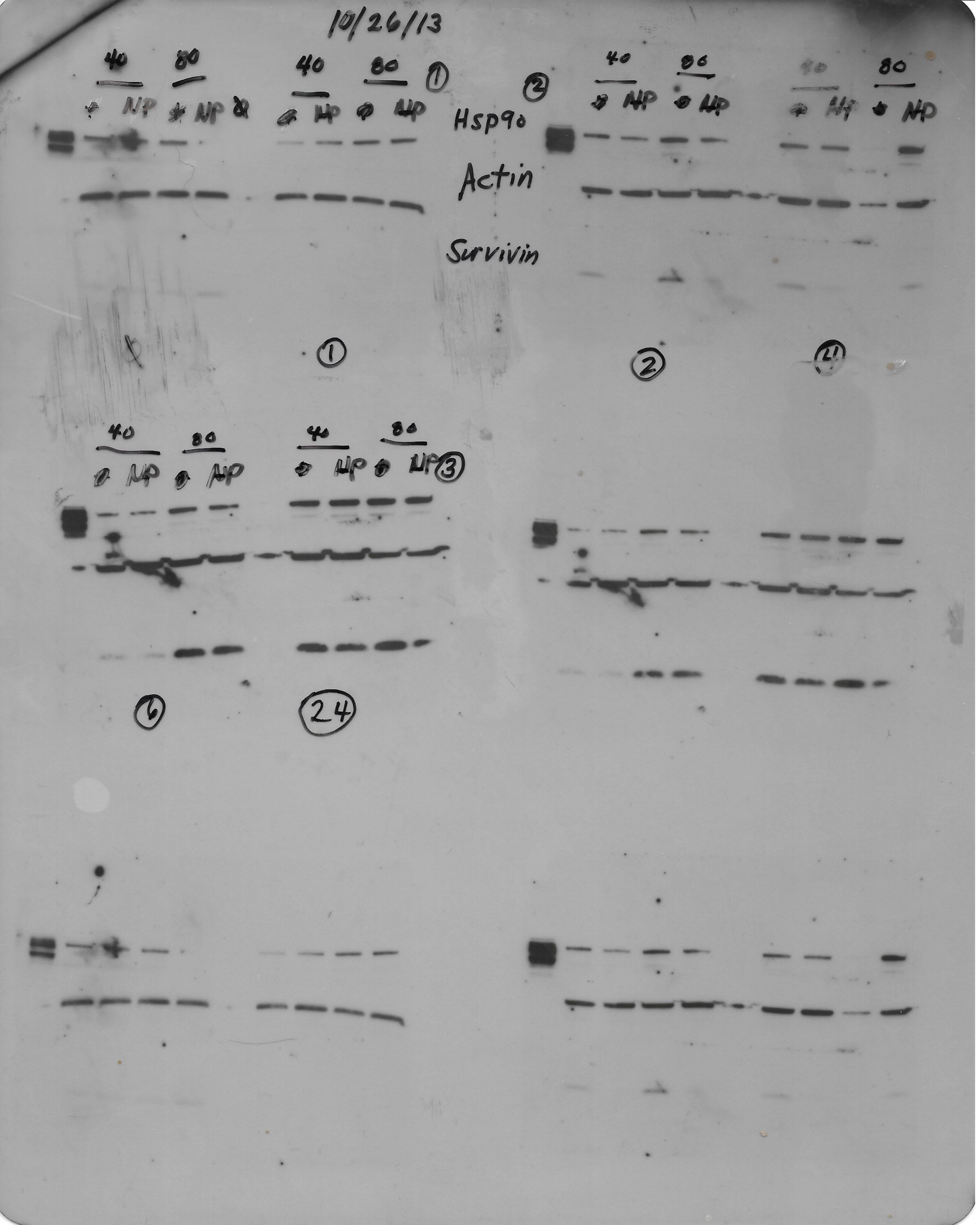

LEFT Three WB different exposure times

Top left 0 and 1h incubation of nanoparticles in cells
Top right 2 and 4h incubation
Middle left 6 and 24h incubation

Each WB membrane was separated into three stipes to probe
Hsp90
Actin
BIRC5

In this series of exposure times only Actin is good in all samples
BIRC5 exposure was good only for 6 and 24 blot

RIGHT Three WB additional exposure times
BIRC 5 exposure good for
WB for 0 and 1h
WB for 2 and 4h

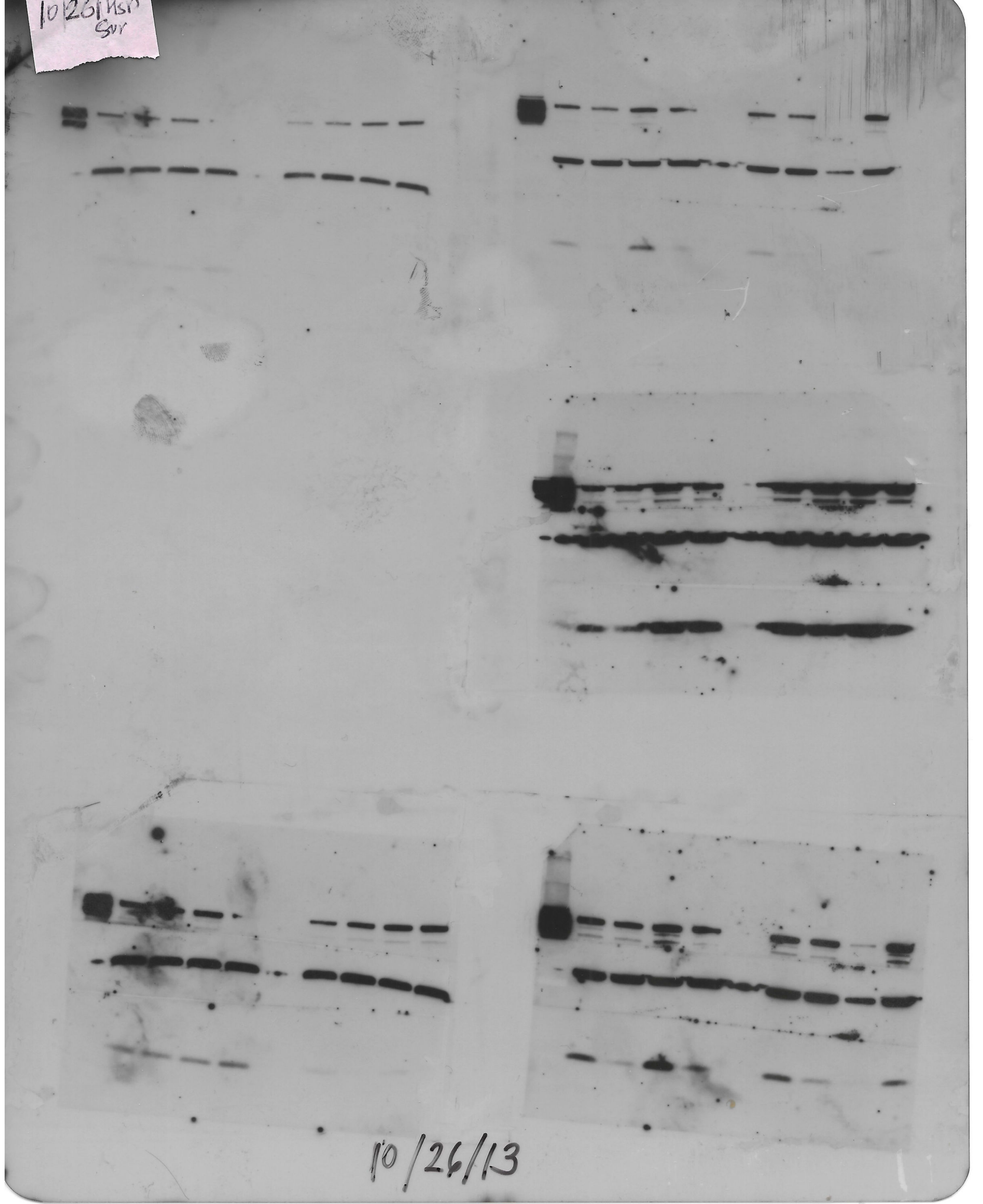

Supplement: Supplementary file 1 [file cancers-13-04497-s001.zip › Western Blot Information/WBs from supernatantas of nanoparticles treated cells - A series.pdf]
