# Supplementary material for: Development of Multi-Scale X-ray Fluorescence Tomography for Examination of Nanocomposite-Treated Biological Samples
Source: Cancers (Basel). 2021 Sep 6;13(17):4497. doi: 10.3390/cancers13174497 (PMC8430782; doi:10.3390/cancers13174497)
Supplement: Supplementary file 1 [file WBs from supernatantas of nanoparticles treated cells - C series.pdf › Western Blot Information/WBs from supernatantas of nanoparticles treated cells - C series.pdf]

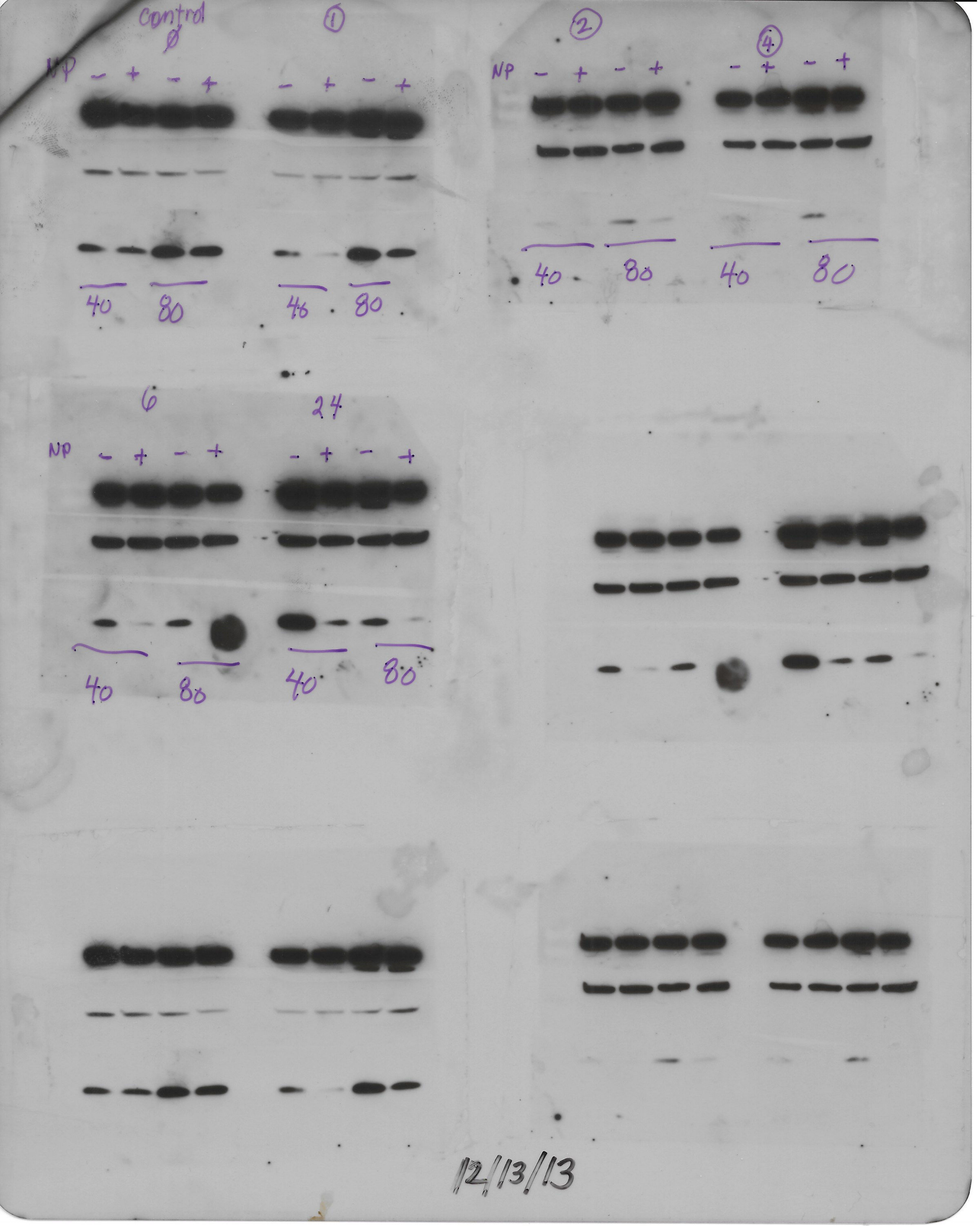


Three WB different exposure times

Top left 0 and 1h incubation of nanoparticles in cells
Top right 2 and 4h incubation
Middle left 6 and 24h incubation

Each WB membrane was separated into three stipes to probe
Hsp90
Actin
BIRC5
